# Functional Coupling between HIV-1 Integrase and the SWI/SNF Chromatin Remodeling Complex for Efficient *in vitro* Integration into Stable Nucleosomes

**DOI:** 10.1371/journal.ppat.1001280

**Published:** 2011-02-10

**Authors:** Paul Lesbats, Yair Botbol, Guillaume Chevereau, Cédric Vaillant, Christina Calmels, Alain Arneodo, Marie-Line Andreola, Marc Lavigne, Vincent Parissi

**Affiliations:** 1 Laboratoire MCMP, UMR 5234 CNRS-Université Victor Segalen Bordeaux 2, Bordeaux, France; 2 Institut Pasteur, UMR 3015 CNRS, Paris, France; 3 Laboratoire Joliot-Curie, USR3010, ENS de Lyon, Lyon, France; University of Geneva, Switzerland

## Abstract

Establishment of stable HIV-1 infection requires the efficient integration of the retroviral genome into the host DNA. The molecular mechanism underlying the control of this process by the chromatin structure has not yet been elucidated. We show here that stably associated nucleosomes strongly inhibit *in vitro* two viral-end integration by decreasing the accessibility of DNA to integrase. Remodeling of the chromatinized template by the SWI/SNF complex, whose INI1 major component interacts with IN, restores and redirects the full-site integration into the stable nucleosome region. These effects are not observed after remodeling by other human remodeling factors such as SNF2H or BRG1 lacking the integrase binding protein INI1. This suggests that the restoration process depends on the direct interaction between IN and the whole SWI/SNF complex, supporting a functional coupling between the remodeling and integration complexes. Furthermore, *in silico* comparison between more than 40,000 non-redundant cellular integration sites selected from literature and nucleosome occupancy predictions also supports that HIV-1 integration is promoted in the genomic region of weaker intrinsic nucleosome density in the infected cell. Our data indicate that some chromatin structures can be refractory for integration and that coupling between nucleosome remodeling and HIV-1 integration is required to overcome this natural barrier.

## Author Summary

The infection and pathogenicity of HIV-1 requires the integration of a DNA copy of its genome into host cell chromosomes. This leads to a stable association between the host and the retrovirus, preventing its total eradication from the patient and the constant archiving of drug-resistant viruses. Even if the reaction mechanism catalyzed by the viral integrase is now well known, its interaction with the host chromatin is incompletely understood. Chromatin is highly structured due to DNA compaction in nucleosomes where the DNA accessibility can affect both the efficiency and the selectivity of integration. Using an *in vitro* assay allowing us to reproduce and monitor easily the integration into an acceptor chromatinized plasmid, we show that the presence of a stable and organized nucleosome prevents the integration of the viral DNA. Additionally, we report that the integration into nucleosome regions can be restored if it is coupled with cellular factors that remodel the chromatin structure. Our results indicate a strong functional interaction between the HIV-1 integration complex and the chromatin maintenance host machinery required for efficient integration of the HIV-1 genome into the cellular DNA. This suggests potentially new therapeutical targets for inhibiting HIV-1 replication, and also potentially new ways for modulating the selectivity of the lentiviral vector-mediated integration in gene therapy.

## Introduction

The integration of viral DNA into the cellular genome is a key mechanism for the establishment of stable HIV-1 infection. This multi-step mechanism catalyzed by the retroviral integrase (IN) is divided into the 3′processing of two viral DNA ends followed by their strand transfer into the target DNA. These reactions can be reproduced *in vitro* independently [1]–[3] or in concerted integration [4]–[6]. However, conditions in cells differ in numerous ways from these *in vitro* reactions. For example, in infected cells, IN is associated with other viral and cellular factors and the viral DNA in a large nucleoprotein complex called the pre-integration complex (PIC) [7]. Furthermore, target DNA is ensconced within chromatin, a highly structured nucleoprotein complex that modulates DNA accessibility to various nuclear proteins or enzymes and can thus affect the efficiency and selectivity of retroviral integration [8]–[12]. Retroviruses target different regions of the genome. For example, HIV-1 preferentially integrates into active genes [13], [14] in contrast to Mo-MLV and ASLV, which displays less preference for active genes containing regions [15], [16]. The effects of the chromatin structure on integration are thought to be responsible for the different selectivity of retroviral integrases. This was confirmed by the finding that DNA compaction by histone H1 affected differently the *in vitro* one-end integration catalyzed by HIV and ASV INs [17]. Functional studies show that the integrase protein appears to be the principle viral determinant responsible for this differential DNA targeting during integration [18]. More recent massive analyses of retroviral DNA integration using the pyrosequencing method better described the cellular chromatin landscape of the integration loci [12], [19], [20]. Those studies indicate that integration is particularly promoted near transcription-associated histone modifications but was not promoted in regions rich in transcription-inhibiting modifications. This property of HIV-1 IN to integrate fairly equally into the active gene, in addition to the integration sites profile observed in infected cells, suggests a link between IN and the cellular chromatin machinery. This is supported by the fact that several well described IN cofactors, as INI1 and LEDGF/p75, belong to the chromatin associated proteins family. INI1, is a core component of the chromatin remodeling complex SWI/SNF that directly interacts with HIV-1 IN [21], either stimulates or inhibits *in vitro* integration and modulates several steps of the HIV-1 replication in cells [22]–[24]. The lens epithelium-derived growth factor (LEDGF/p75) is another major IN cofactor essential for efficient retroviral replication [15], [25]–[27] and involved in the choice of integration sites [15], [28], [29]. Interestingly, affecting the selectivity of LEDGF/p75 can modify the selectivity of HIV-1 IN and provide new tools for gene therapy lentiviral vectors [30]–[33].

Taken together all those data support a functional interaction between the retroviral integration machinery and cellular factors modifying or sensing histone modifications associated with the transcription process. However, the molecular mechanism underlying the efficient integration into the chromatin structure has not yet been elucidated. An *in vitro* integration assay using linear polynucleosomes as acceptor templates made it possible to study the effect of chromatin on half-site one-end integration (HSI) and the regulating function of LEDGF/p75 [34]. However, it was not possible to determine the impact of chromatin on the physiological two viral-end full-site integration reaction (FSI) undetectable in this system. To analyze both the efficiency and the selectivity of HIV-1 integration into chromatin, we used a circular chromatinized substrate to detect both FSI and HSI forms. We report here for the first time that, in contrast to HSI, efficient FSI integration is disfavored in areas of stable and regularly positioned nucleosomes, and that chromatin remodeling of these domains mediated by the SWI/SNF complex containing IN cofactor INI-1 is required to overcome this barrier.

## Results

### *In vitro* assembled nucleosomes array is refractory to full-site concerted integration but not half-site integration

**Figure 1:**
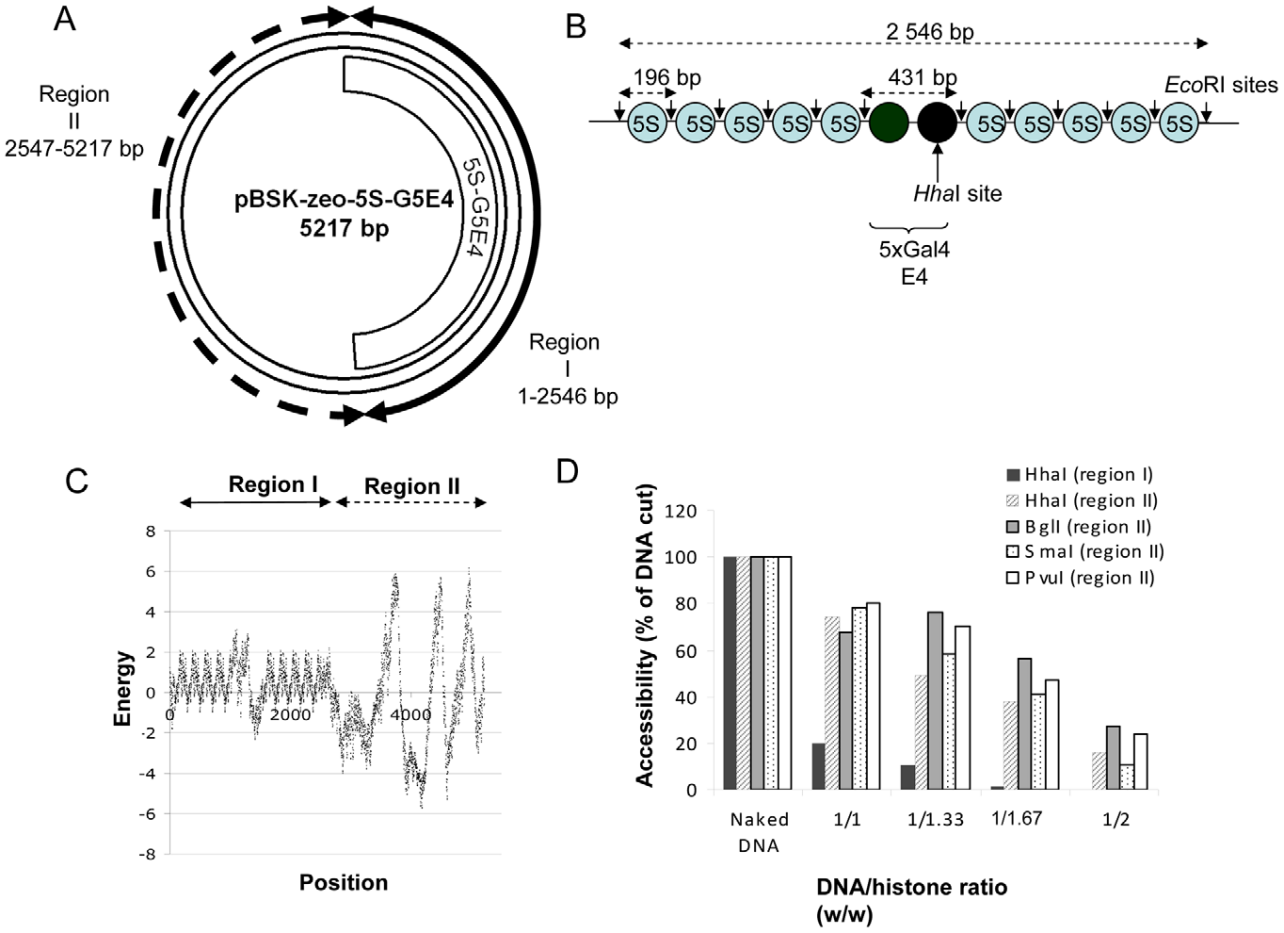
Structure of the pBSK-zeo-5S-G5E4 chromatinized acceptor DNA. The pBSK-zeo-5S-G5E4 vector (**A**) contains the previously described 5S-G5E4 phasing region (**B**) and an outer region without phasing sequence (pBSK-zeo backbone) containing the *Sh ble* gene and EM-7 promoter. The grey ovals represent the 5S RNA stably positioned nucleosomes, black ovals represent two nucleosomes covering five Gal 4 binding sites and adenovirus 2 E4 minimal promoter. Arrows show the *EcoR*I sites containing regions. Nucleosome stability and positioning on the vector was predicted using the model described by [39] (**C**). The energy of nucleosome formation was plotted for each position of the template sequence. Two regions were determined: region I containing stably and regularly associated nucleosomes and region II with more labile nucleosomes. The structure of the two regions was further checked by REA assay (**D**) using enzymes cleaving in region I, II or both and the percentage of cleavage was reported as the result of a typical representative experiment.

The circular chromatinized acceptor pBSK-Zeo-5S-G5E4 vector described in Figure 1A was constructed by cloning the 5S-G5E4 nucleosome positioning sequence reported in Figure 1B into the previously described pBSK-Zeo backbone [35]. The 5S-G5E4 fragment contains 2×5 nucleosome positioning sequences (5S) that allow the *in vitro* assembly of a stable and regularly spaced polynucleosome (PN) template. This PN has been extensively used to study chromatin remodeling, histone modifications and several enzymatic processes occurring on chromatin *in vitro* [36]–[38]. The structure of the chromatinized plasmid was first predicted *in silico* using the nucleosome occupancy prediction algorithm described in [39] and derived from [40]. The analysis reported in Figure 1C suggests that the circular PN assembled on the pBSK-Zeo-5S-G5E4 vector contains two different chromatin regions: stably positioned and regularly spaced nucleosomes in region I and less dense and more dynamic nucleosomes in region II. The formation of two distinct chromatin structures was experimentally confirmed by typical restriction enzyme assay (REA) using enzymes cleaving in region I, region II or in both regions (cf restriction site positions in Figure S1A). Results showed highly reproducible differences in restriction site accessibility in both regions after nucleosome assembly (Figure 1D). Correct positioning of the nucleosomes on in the 5S-G5E4 region was further controlled by specific *Eco*RI cleavage of the region followed by agarose gel shift allowing to precisely detect mono- and di-nucleosomes on well assembled templates (Figure S1B). The naked and plasmid PN assembled with increasing DNA/histones were therefore used as acceptor templates in concerted integration assays in order to determine the effect of these chromatin structures on the selectivity and the efficiency of both half-site and full-site integration.

**Figure 2:**
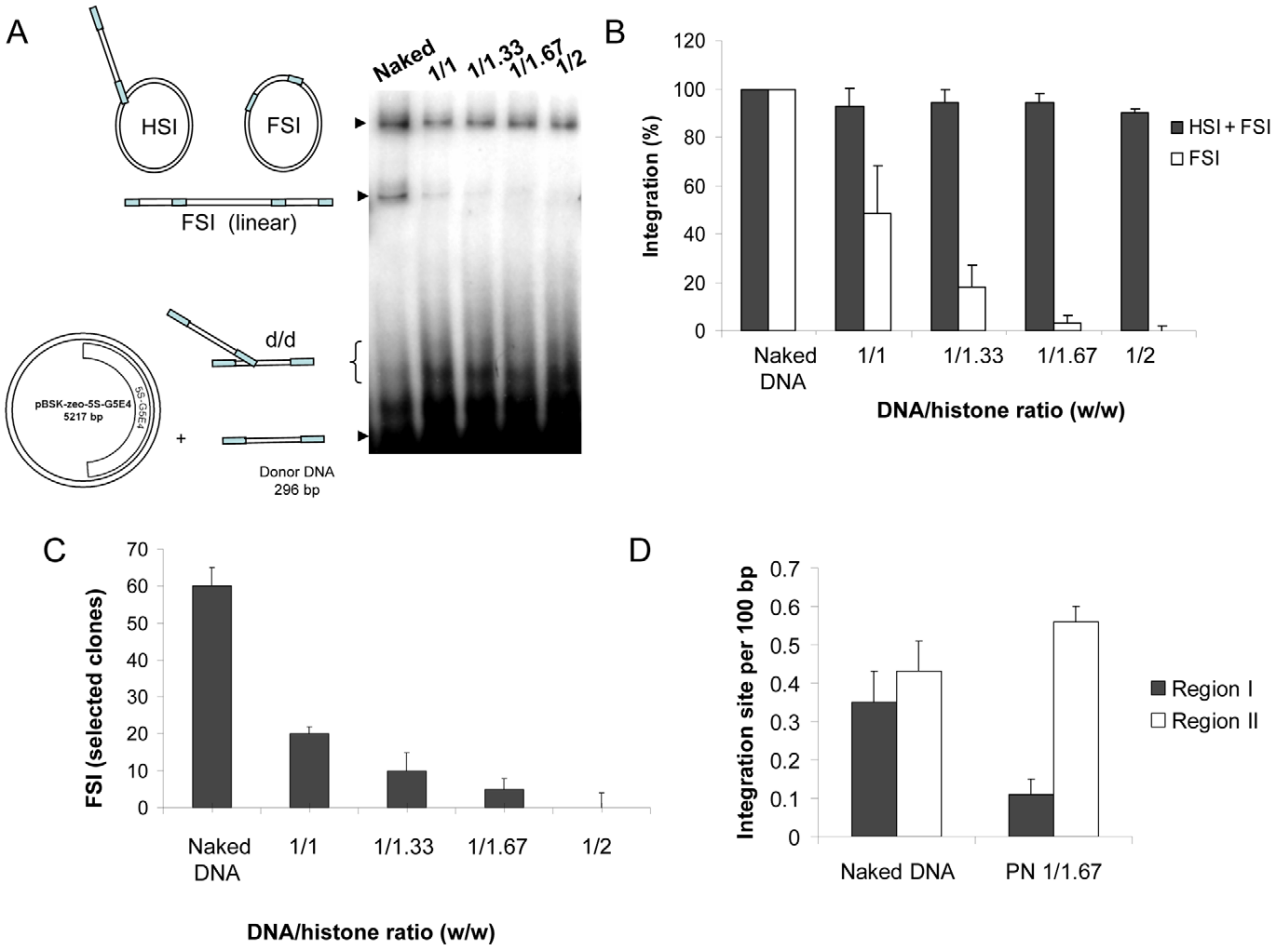
Effect of nucleosome assembly on *in vitro* HIV-1 integration. A concerted integration assay was performed with 12 pmoles of IN and 10 ng of donor DNA and 100 ng of naked pBSK-zeo-5S-G5EA plasmid (Naked), or polynucleosomal pBSK-zeo-5S-G5E4 assembled with increasing amounts of histones expressed in DNA/histones mass ratio (µg/µg) (1/1, 1/33, 1/167, 1/2). The reaction products were loaded on 1% agarose gel (**A**). The position and structures of the donor substrate and different products obtained after half-site (HSI), full-site (FSI) and donor/donor integration (d/d) are shown. The different integration products were quantified by densitometric estimation of the FSI and HSI+FSI heterointegration bands with the Image J software (**B**). The circular FSI products were specifically quantified by cloning in bacteria and reported as the number of ampicillin-, kanamycin- and tetracycline-resistant selected clones (**C**). FSI products obtained after integration into the naked or PN 1/1.67 vector were sequenced and 20 correct integration loci were localized in the vector sequence. Results are reported as number of integrations per 100 bp of DNA for region I containing the stably assembled nucleosomes and region II carrying more labile nucleosomes (**D**). All the values correspond to the mean ± standard deviation (error bars) of three independent sets of experiments.

Integration assays were performed using the unprocessed SupF donor DNA and the naked or PN pBSK-zeo-5S-G5E4 templates. Experimental procedure and analysis of the integration products were performed as previously reported [35] (the result of a typical concerted integration reaction is provided in Figure S2). We first observed a large DNA/histone ratio-dependent inhibition of the linear FSI products formed on the PN templates compared to the FSI products formed on the naked vector (Figure 2A and quantification in 2B). In contrast, the circular HSI+FSI products were less affected by the PN assembly. The cloning and the specific quantification of the circular FSI integration products showed that FSI efficiency was severely impaired on the PN substrate (Figure 2C). No significant change in the fidelity of the integration reaction was detected for each condition, as reported in the integration loci structure determination by sequencing (Figure S3). Data were highly reproducible on multiple independent sets of chromatinized templates. However, the inhibition efficiency did vary from one set to another depending on the efficiency of the nucleosome positioning on the template controlled by restriction analysis reported in Figure 1D and Figure S1B. Control experiments performed with purified histones added in standard integration reaction solutions did not show any significant inhibition except using excess amounts of protein (data not shown) indicating that the inhibition observed with the PN template was not due to the free unbound histones remaining in the solution after assembly. This was confirmed by the results of integration assays performed with the pBSK-zeo vector lacking the 5S-G5E4 sequence and assembled with the same amounts of histones (checked by typical REA assay). Indeed, in this case, no inhibition was found as it would be expected if due to free histones (Figure S4).

In order to determine whether this inhibition profile was dependent on the way the nucleosomes were assembled on the templates we tested other differently assembled PN on integration. An assembly method based on the use of Acf1/ISWI complex in presence of recombinant histone chaperone NAP-1 and topoisomerase was chosen since in this case the nucleosome positioning mechanism is highly different from the one followed by salt dialysis [41]. As checked by REA assay (Figure S5A) with this system 1/0.1 to 1/0.6 DNA/histone ratios (w/w) could be efficiently assembled. The same integration inhibition effect of the nucleosomes was observed using these new PN (Figure S5B) indicating that positioned nucleosomes inhibited *in vitro* integration on DNA templates independently from the assembly method. The salt dialysis method was chosen for the next steps of study because of its better compatibility with extensive analyses and the absence of assembly factor that could interfere with the following integration reaction.

### Stable and regularly spaced nucleosomes strongly inhibit full-site integration *in vitro*

The integration inhibition observed in PN templates could result from modifications in the DNA topology of the receptor plasmid. To test this hypothesis, the PN templates were relaxed by Topoisomerase I before to be used as acceptor substrates. As shown in Figure S6, no drastic change in the global inhibition profile was observed after Topoisomerase I treatment. This confirmed the results obtained in control experiments using the assembled pBSK-zeo vector. Indeed when integration was performed on these templates no significant inhibition of both HSI and FSI was detected (see Figure S4). Taken together, our data led us to conclude that the inhibition of concerted integration by the nucleosomes was mainly due to the stable nucleosomes assembled in region I (absent in pBSK-ZEo vector) and not to massive changes in DNA topology of the chromatinized plasmids (expected to occur at a similar level in pBSK-zeo and pBSK-zeo-5S-G5E4 chromatinized vectors).

If physical protection of the DNA by the nucleosomes is responsible for the inhibition of FSI integration in region I, then we should observe a correlation between the density of integration sites and the accessibility of restriction sites throughout the chromatinized vector (reported in Figure 1D). Thus, a greater protection of integration is expected in region I compared to region II. To elucidate this point, integration loci found in the circular FSI products obtained on the PN pBSK-zeo-5S-G5E4 template were sequenced. A PN acceptor template assembled with a 1/1.67 DNA/histone ratio (w/w) was chosen since enough integrants could be selected in bacteria for further analysis, even if strong inhibition of FSI was observed under these conditions (Figure 2C). Under these conditions, in contrast to the corresponding naked DNA vector, a large redistribution of the integration loci outside the 5S-G5E4 region I was observed (Figure 2D and integration loci localization in Figure S7).

### Treatment of PN templates with the human SWI/SNF chromatin remodeling complex restores and re-directs full-site integration into the dense nucleosome region

Since the DNA protection by the nucleosome was probably responsible for the integration refractory property of region I, we wondered whether nucleosome remodeling could restore integration into the stable chromatin domain.

Remodeling of nucleosomes can be conducted by several protein complexes in the cell (for a review see [42]). We focused here on the human SWI/SNF complex since a major component of this complex, INI1, interacts with HIV-1 IN and is thought to regulate its activity both *in vitro* and in functional cellular assays [21], [43]–[45]. The human SWI/SNF complex was purified from HeLa cells following well defined a previously published purification protocols allowing to obtain an active complex without major contaminants [46]. The remodeling activity of the purified complex was checked on our chromatinized integration templates under conditions described previously [47] using REA assay (S8A).

**Figure 3:**
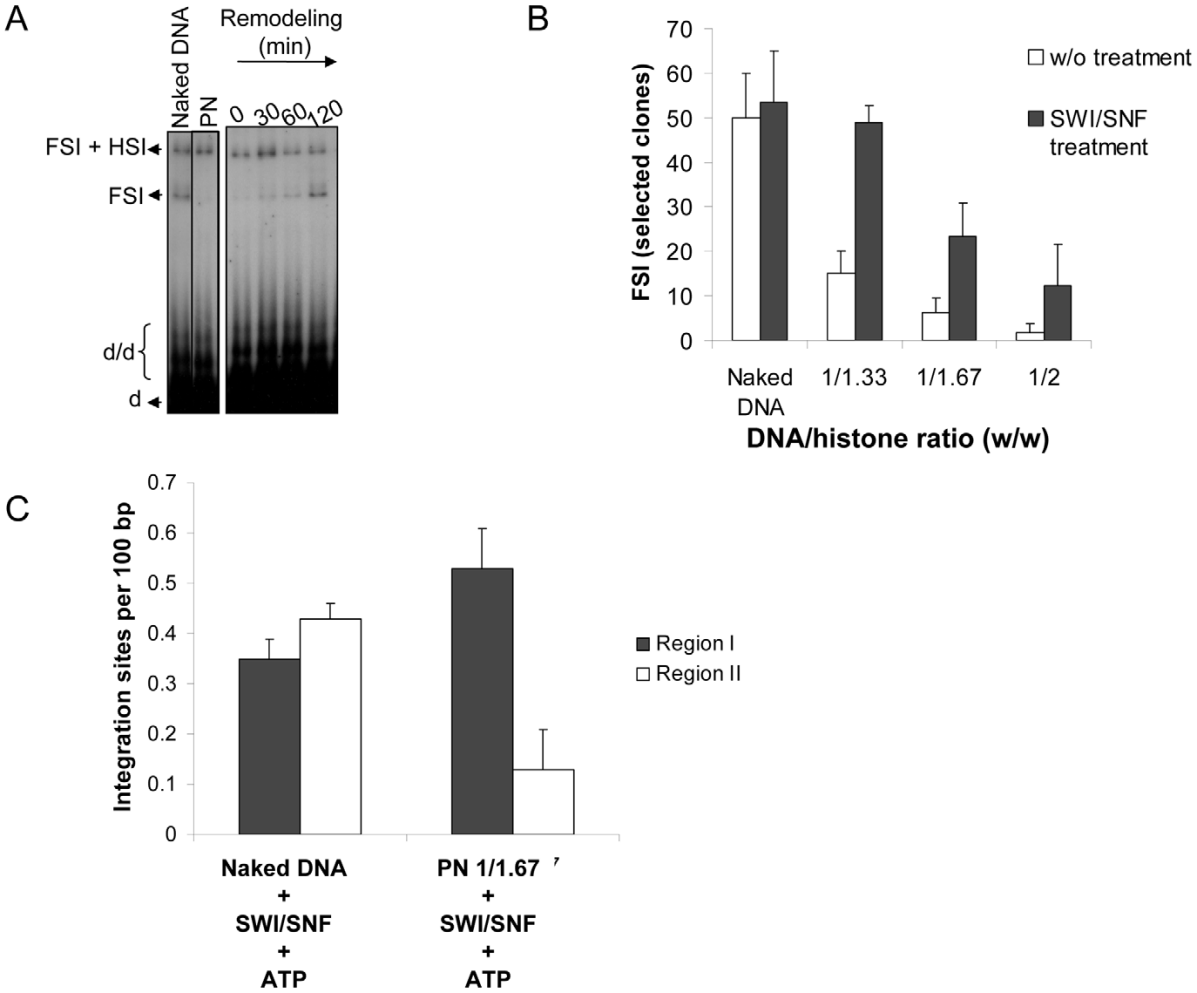
Effect of chromatin remodeling activity of the SWI/SNF complex on the *in vitro* integration in nucleosomal templates. Naked or chromatinized pBSK-zeo-5S-G5EA vector was used as acceptor substrate in a concerted integration assay performed after SWI/SNF treatment in presence of ATP (0 to 120 minutes) with 12 pmoles IN, 10 ng donor and 100 ng acceptor. Efficient remodeling of the vector was previously checked by REA assay (see Figure S6A). The reaction products were loaded on 1% agarose gel and an example of the result obtained with the 1/1.67 PN template is reported in (**A**). The position the different products obtained after half-site (HSI), full-site (FSI) and donor/donor integration (d/d) are shown in addition to donor (d). The circular FSI products obtained with vectors assembled with increasing amounts of histones expressed in DNA/histones mass ratio (µg/µg) (1/33, 1/167, 1/2) with or without SWI/SNF pre-treatment were specifically quantified by cloning in bacteria and reported as the number of ampicillin-, kanamycin- and tetracycline-resistant selected clones (**B**). 20 correct integration loci were localized in the vector sequence and the number of integration events obtained with the naked or 1/1.67 PN template remodeled by SWI/SNF was quantified and is shown as integration number per 100 bp of DNA for region I containing the stably assembled nucleosomes and region II carrying more labile nucleosomes (**C**). All the values are the mean ± standard deviation (error bars) of three independent sets of experiments.

To determine the effect of this remodeling activity on concerted integration, both reactions were coupled. Briefly, acceptor plasmids were treated with the purified SWI/SNF complex and then IN was added to the reaction solution under conditions allowing the integration reaction. As shown in Figure 3A, remodeling of the PN templates by SWI/SNF led to recovery of full-site integration on these templates. Same results were obtained with the other PN (S8B). Quantification of the circular FSI products in bacteria confirmed the integration recovery after SWI/SNF treatment (Figure 3B). Additional gel filtration purification of the SWI/SNF complex followed by the assay of the eluted fractions in IN-mediated concerted integration into PN showed that the integration restoration activity co-purified with the active remodeling complex. This led us to rule out the possible contaminant source for the restoration effect (data not shown).

Sequencing of the integration loci was then performed in order to analyze the effect of SWI/SNF remodeling on the selectivity of FSI. We found that remodeling of the PN assembled on the pBSK-zeo-5S-G5E4 vector was responsible for a global restoration of FSI integration and an important re-direction of the integration events into region I (Figure 3C). On the other hand, there was no change in integration fidelity (Figure S8C). In contrast, incubation of the naked pBSK-zeo-5S-G5E4 vector with the SWI/SNF remodeling complex did not change the random distribution of the integration sites (shown in Figure 2D) and the proportion of integration sites in region I of this vector was not increased. This result confirms that the restoration of integration within the 5S-G5E54 region after remodeling requires its chromatinization. The molecular mechanism underlying this change of integrase selectivity was then studied.

### Full-site integration into stable nucleosomes requires the whole active SWI/SNF complex

**Figure 4:**
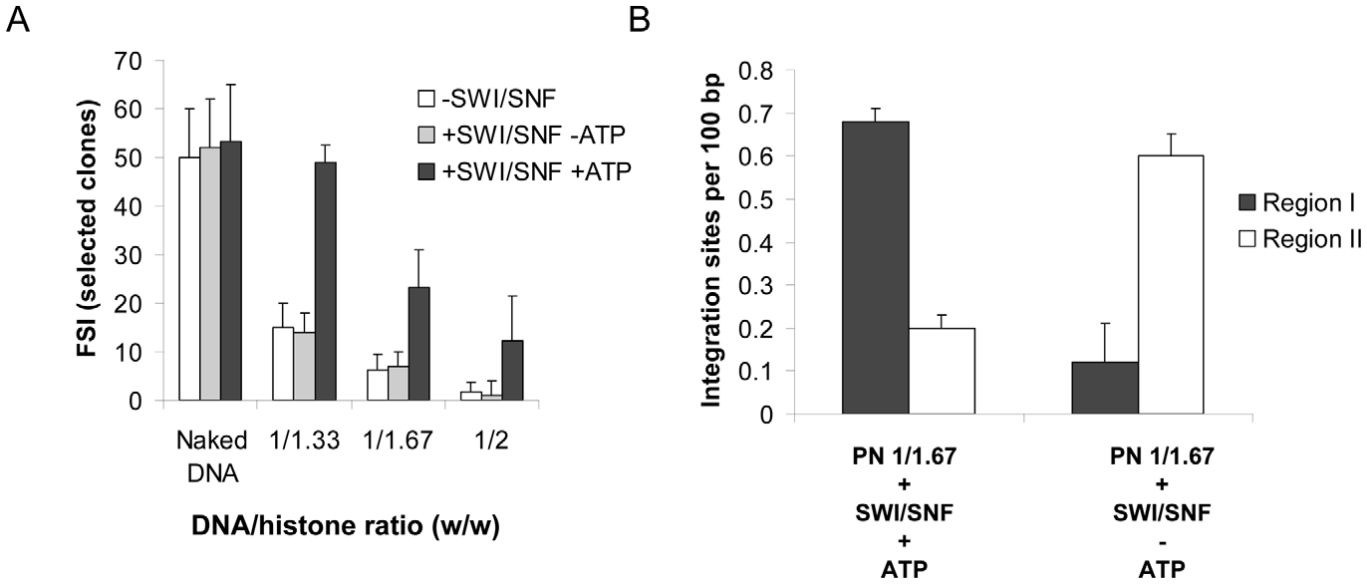
Effect of ATP on the *in vitro* integration restoration and targeting property of the SWI/SNF complex. A concerted integration assay was performed with 12 pmoles of IN and 10 ng of donor DNA and 100 ng of naked or PN pBSK-zeo-5S-G5E4 treated or not for 2 hours with SWI/SNF complex in presence or absence of ATP. The circular FSI reaction products obtained in each condition were specifically cloned and quantified in bacteria as the number of ampicillin-, kanamycin- and tetracycline-resistant selected clones (**A**). 20 correct integration loci were localized in the vector sequence and the number of integration events obtained with the PN 1/1.67 vector treated with SWI/SNF in presence or absence of ATP were sequenced and reported as integration number per 100 bp of DNA for region I containing stable nucleosomes and region II containing more labile nucleosomes (**B**). Values correspond to the mean ± standard deviation (error bars) of three independent experiments.

We first tested whether the remodeling activity of the SWI/SNF complex was required for both integration restoration and targeting into stable nucleosomes. As known and confirmed in the REA assay reported in Figure S8A, the remodeling activity of this complex requires ATP. When integration experiments were performed using PN templates treated with SWI/SNF in the absence of ATP, neither the restoration of integration (Figure 4A) nor its targeting towards region I (Figure 4B and integration loci localization in Figure S9) was observed. This result points out the ATP dependence of the integration restoration and strongly suggests that SWI/SNF nucleosome remodeling activity is required for the process.

**Figure 5:**
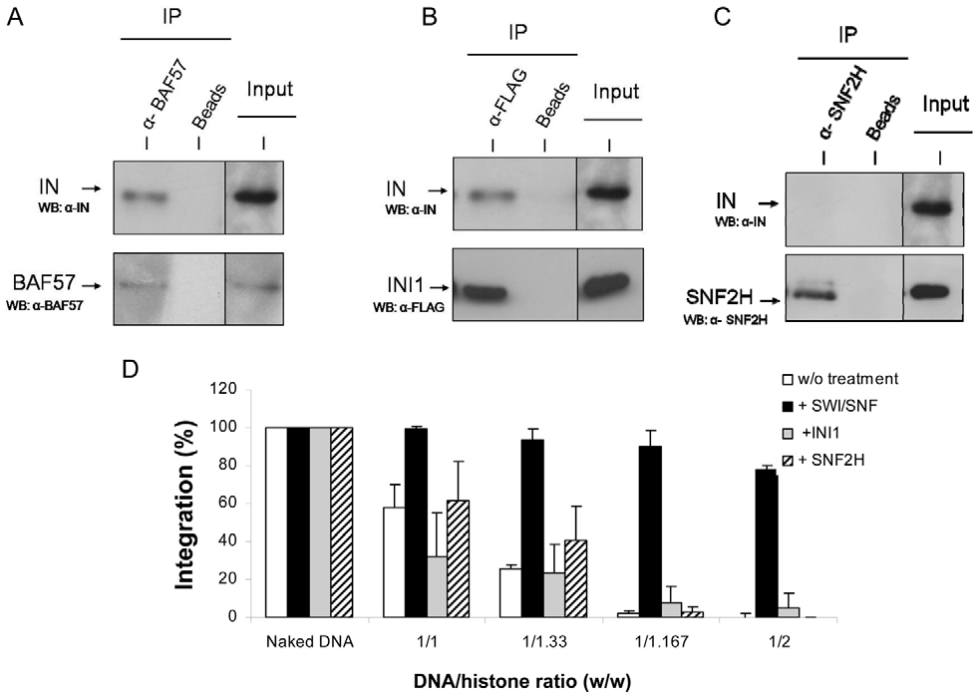
Physical and functional *in vitro* interaction between HIV-1 IN and human SWI/SNF complex, INI1 protein or SNF2h. Recombinant pure HIV-1 IN and SWI/SNF complex were subjected to co-immunoprecipitation analysis using α-BAF57 antibodies (**A**). Immunoprecipitation without antibody was used as control (Beads). The initial amount of protein used is shown (Input). Recombinant pure HIV-1 integrase and FLAG-tagged INI1 were subjected to immunoprecipitation using α-FLAG or no antibodies (Beads) (**B**). Recombinant pure HIV-1 integrase and FLAG-tagged INI1 were subjected to immunoprecipitation using α-FLAG or no antibodies (Beads). Recombinant pure HIV-1 integrase and FLAG-tagged INI1 were subjected to immunoprecipitation using α-FLAG or no antibodies (Beads). Bound proteins and input controls were immunoblotted with α-BAF57, α-FLAG or α-IN antibodies. Recombinant pure HIV-1 integrase and SNF2H proteins were subjected to co-immunoprecipitation using α-SNF2H or no antibody as control (Beads) (**C**). Initial amount of protein used is shown (Input). Bound proteins and input controls were immunoblotted with α-SNF2H or α-IN antibodies. In all panels, WB =  Western blot analysis. Functional interactions were studied in a concerted integration assay performed with 12 pmoles of IN and 10 ng of donor DNA and 100 ng of naked or PN pBSK-zeo-5S-G5E4 assembled with increasing amounts of histones expressed in DNA/histones mass ratio (µg/µg) (1/33, 1/167, 1/2) treated or not for 2 hours with SWI/SNF complex or FLAG-tagged INI1 under the same conditions in presence of ATP (**D**). The FSI products were quantified by densitometric estimation of the linear FSI bands with the Image J software and reported as percentage of integration. Values correspond to the mean ± standard deviation (error bars) of at least three independent experiments.

Owing to the direct interaction between IN and INI1, a component of the SWI/SNF complex [21], the most reasonable hypothesis explaining our observations would be a direct interaction between IN and the active SWI/SNF complex, leading to a functional coupling between its nucleosome remodeling property and the integration reaction. *In vitro* immunoprecipitation between the recombinant purified IN and the SWI/SNF complex (Figure 5A) supported this hypothesis and led us to assume that the binding of IN to the remodeling complex occurred via the IN•INI1 interaction reported above and also observed in our conditions (Figure 5B). To determine whether this interaction was sufficient for the restoration and targeting process, we tested the effect of the isolated INI1 factor on concerted integration into PN substrates. As shown in Figure 5D, treatment of the PN substrate with INI1 did not make it possible to overcome the inhibitory effect of the nucleosomes, as observed with the entire remodeling complex. Therefore, the IN•INI1 interaction was not sufficient to restore an efficient concerted integration into the chromatinized template confirming the requirement of a coupling between integration and the remodeling activity.

### Restoration of integration into stable nucleosomes involves the direct binding of IN to SWI/SNF complex

To determine whether this coupling needs a direct interaction between HIV-1 IN and the remodeling complex we first tested another human remodeling enzyme that doesn't bind IN. The SNF2H enzyme was chosen since it is the catalytic subunit of the human ACF/SNF2H chromatin remodeling complex, another main human chromatin remodeling complex [48] and no direct *in vitro* interaction was found between the purified SNF2H and HIV-1 IN in immunoprecipitation experiments (Figure 5C). Additionally the remodeling efficiency of SNF2H on the pBSK-zeo-5S-G5E4 PN templates was found similar to the one detected for SWI/SNF (see Figure S10). Thus we tested the effect of SNF2H remodeling on integration restoration into PN templates and compared it with the one detected with SWI/SNF. Integration experiments showed that, despite the capability of SNH2H to remodel the PN template, no restoration of activity was observed after this treatment in contrast to SWI/SNF (Figure 5D). However the lack of restoration with SNF2H could be due to some differences in the remodeling process catalyzed by this factor comparing to SWI/SNF [49], [50]. To better address the specificity of the restoration observed with SWI/SNF and to determine the role of IN•SWI/SNF interaction in this mechanism, we tested the isolated catalytic BRG1 subunit of this complex which was previously shown to possess a remaining remodeling activity [50] but lacks the integrase binding INI1 cofactor (no direct interaction between HIV-1 IN and BRG1 protein was detected under our immunoprecipitation conditions).

**Figure 6:**
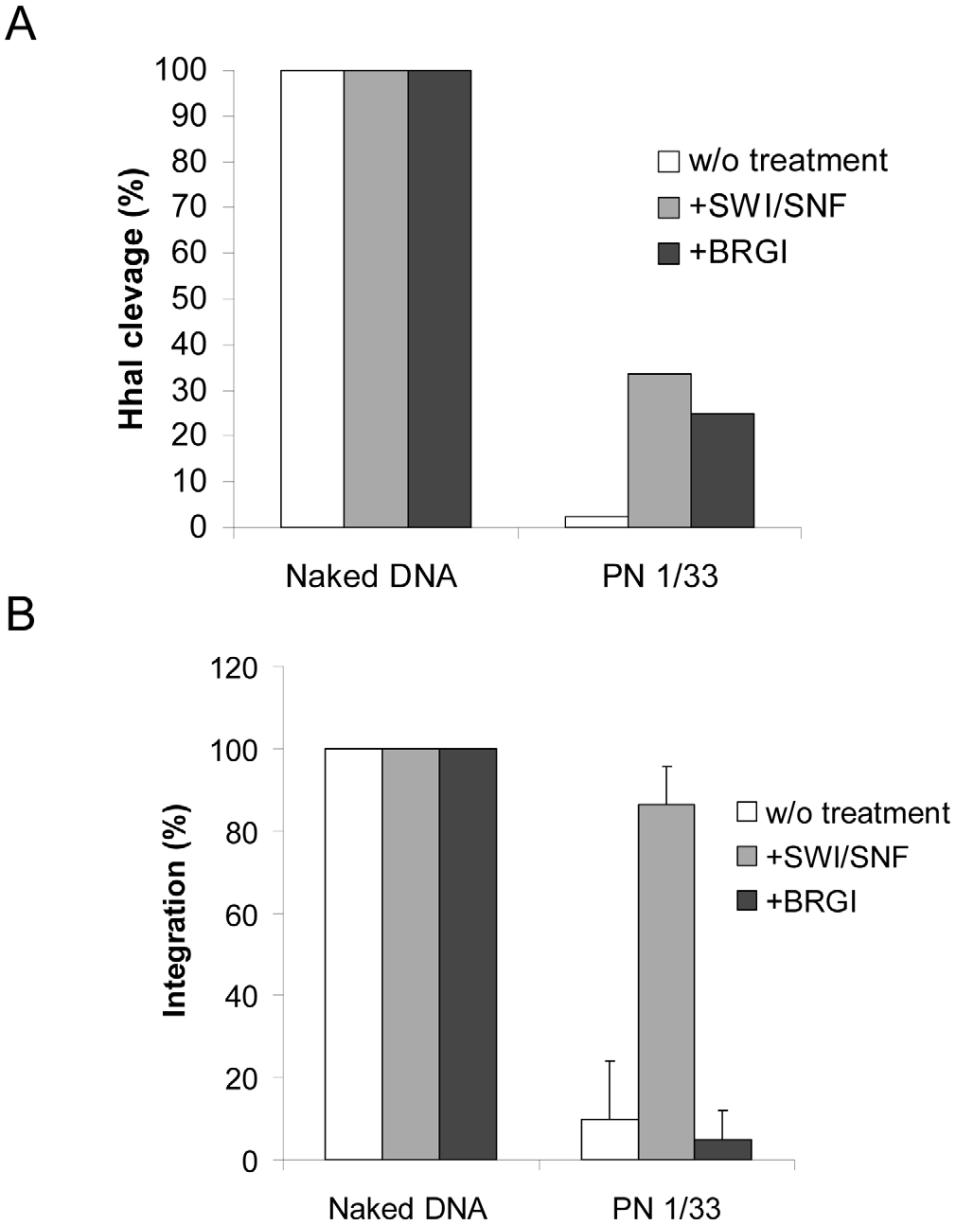
Comparison between the effect of nucleosome remodeling by BRG1 or SWI/SNF on *in vitro* integration into PN. Naked or 1/1.33 DNA/histones mass ratio (µg/µg) chromatinized pBSK-zeo-5S-G5EA vectors were treated with 0.6 pmoles of BRG1 for two hours. The remodeling efficiency was controlled in a REA assay (here *Hha*I restriction) (**A**). Naked or chromatinized vector were used as acceptor substrate in a standard concerted integration assay performed without remodeling treatment or after SWI/SNF or BRGI. Heterointegration products were quantified on agarose gel using the Image J software and reported as percentage of integration. The results of a typical representative REA experiment is shown in **A** and the data are reported as percentage of DNA cleaved. The means of three *in vitro* integration experiments were reported in **B** and data were compared to a 100% of integration corresponding to the standard integration conditions (naked DNA without treatment).

We first checked the remodeling activity of BRGI on our PN templates. Our remodeling assays indicated that even if BRGI displayed similar activity than SWI/SNF on linear PN template it was less active on our circular PN at high DNA/histones ratios (>1/1.33, data not shown). However similar remodeling efficiency was found for BRGI and the whole complex at moderate ratios (see Figure 6A). Thus a 1/1.33 DNA/histones ratio was chosen for analyzing the effect of BRG1 remodeling on the *in vitro* integration. As reported in Figure 6B, in contrast to SWI/SNF treatment, no restoration of integration was detected with BRG1 despite the similar remodeling activity found between the two complexes.

This indicates that efficient integration into PN requires both the remodeling activity of SWI/SNF and its direct interaction with HIV-1 IN. This data provides evidence for a model where a direct functional coupling between HIV-1 IN and the entire active SWI/SNF complex is required for efficient *in vitro* integration a nucleosome-dense region. To further evaluate this refractory property of nucleosomes in cells we analyzed the position of several integration sites identified in the genome of infected cells and compared them with the nucleosome positions on the genome.

### HIV-1 integration into transcriptional units of infected cells is favored in less dense nucleosome regions

**Figure 7:**
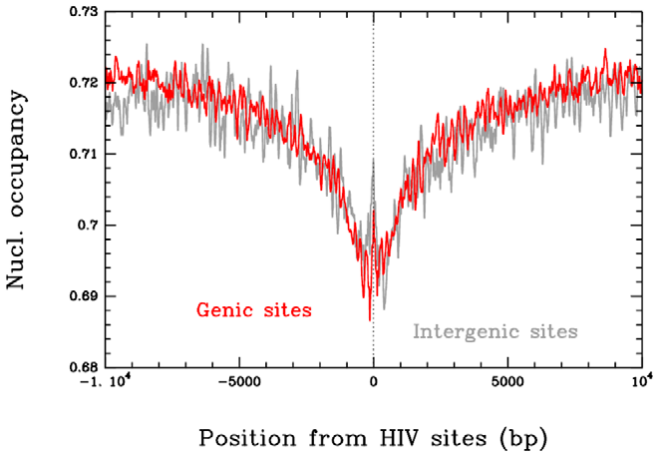
Correlation between *in vivo* HIV-1 integration site and nucleosome occupancy. 41435 integration loci were obtained from [12] and submitted to the nucleosome-positioning prediction analysis set up previously [39]. The predicted high-density (1 nuc./200 bp.) nucleosome occupancy was reported and the position of the integration site was plotted as dotted line. Comparison between the intragenic and intergenic integration sites is reported. Results are the means of the analyses performed from the 41435 integrands. Data correspond to the nucleosome occupancy averaged over integration sites in 9017 intergenic regions (grey) and 32418 genic regions (red).

HIV-1 integration has been shown to be favored in transcriptional units [12], [19], [20]. We further compared the positions of integration sites and nucleosomes in both transcription units and intergenic regions. For this analysis, the sequences surrounding 41 435 non redundant integration sites were selected from previous studies [12] and submitted to a nucleosome-positioning predictive algorithm [39]. The probabilities of nucleosome occupancy were calculated along the 41 435 selected sequences and averaged using the integration sites to align the sequences. The average profile presented in Figure 7 clearly shows that HIV-1 integration sites are mainly found in sequences with lower nucleosome occupancy (0.69) in both genic and intergenic regions as compared to the mean occupancy found at distal sites (0.72 at 10 kbp). This result obtained on a genomic scale with actual HIV-1 integration sites confirms that nucleosome-dense regions are refractory to HIV-1 integration, as observed in our *in vitro* assays of concerted integration into chromatinized templates.

## Discussion

The relationship between HIV-1 integration and cellular DNA structure is poorly documented mostly owing to the lack of efficient *in vitro* systems to address this question. We constructed an *in vitro* acceptor circular DNA substrate containing two differently chromatinized regions: a 5S-G5E4 nucleosome phasing domain (region I) where histones are found in highly dense, stable and regular association with DNA, and an outer domain (region II) where nucleosomes are less dense and irregularly spaced in dynamic complexes. The differential structure of these regions was confirmed both by REA assay and by nucleosome-positioning prediction. Using this template, we analyzed the effect of these two chromatin structures on both HSI and FSI integration events. Quantification of the efficiencies of these activities showed that, unlike HSI, the physiological FSI reaction was severely impaired by nucleosomes. Moreover, the sequencing of the integration loci in the chromatinized plasmid confirmed that the stable nucleosome region I was strongly refractory to two viral-end concerted integration, in contrast to the outer region II containing less stable histones/DNA complexes. Importantly, in all the conditions used in this study, only the efficiency of the integration reaction was affected by nucleosomes in contrast to integration fidelity. This indicates that the nucleosomes did not affect the quaternary structure of the incoming active integration complexes, which was previously shown to strongly impact the fidelity of the integration reaction [51].

This inhibition was found independent of the nucleosomes assembly method. Furthermore, no inhibition of integration was observed in the chromatinized pBSK-zeo lacking the 5S-G5E4 region and the relaxation of the chromatinized pBSK-zeo-5S-G5E4 vector by topoisomerase I, didn't change the efficiency of integration in this PN. Therefore, the inhibition observed on the chromatinized pBSK-zeo-5S-G5E4 vector was due to the stably associated nucleosomes in the 5S-G5E4 region I and not to DNA topology changes induced by nucleosome assembly. In contrast, the outside pBSK-zeo backbone region II containing more labile nucleosomes was less refractory to the integration catalyzed by HIV-1 IN.

These results first appeared contradictory with previously reported data [8], [52], indicating that nucleosomes were preferred target for *in vitro* integration. However substantial differences exist between our present study and these works including the different origin of the analyzed integrases (MLV versus HIV) and their difference in selectivity. Furthermore, even if the two integration mechanisms are similar, some important features diverge: the two proteins do not interact with the same cofactors (no interaction with LEDGF and INI1 were described for MLV) and MLV and HIV do not integrate with the same stagger. This stagger could induce different constraints that, in the case of HIV, could not be compatible with integration into nucleosomes. Finally the works performed previously did not distinguish the partial HSI and the real FSI integration and our data show that the prerequisites for integration into chromatin are not the same for the HSI and FSI reactions.

Indeed, on stable nucleosomes assembled on positioning sequences (region I), we observed a clear difference between the efficiencies of HSI and FSI. This result suggests that the accessibility of the two strands of the DNA helix all along the nucleosome structure, does not fit with the structural requirement for an efficient FSI reaction. The requirement for HSI is different, as shown in several previous studies, and corresponds to phosphodiester bounds located in enlarged DNA major grooves facing out the histone octamer. This difference between HSI and FSI efficiencies on a nucleosome structure is very informative on the catalytic process and suggests a simultaneous strand transfer reaction of the two viral strands in the acceptor DNA. This reaction would be strongly disfavored on a DNA structure closely wrapped around a native nucleosome structure, but, as we will discuss below, could be favored on a remodeled nucleosome.

A recent crystallization of the full length prototype foamy virus integrase and the derived HIV-1 integrase structural model suggests that the binding of a nucleosomal DNA to the intasome containing a tetrameric IN bound to two viral ends would require at least local remodeling for two-end concerted integration ([53] and personal communication from P. Cherepanov). In this model the nucleosome structure could decrease the access and flexibility of the DNA helix necessary for the concerted integration (even if the 4–6 bp stagger is an indicator of major groove recognition). Our full-site integration data obtained on chromatinized templates indicate that a labile chromatin structure is a better substrate for retroviral integration than a stable nucleosome domain. This confirms that a dynamic chromatin structure can allow the loci to adapt for two viral-ends integration, nucleosomes being more easily displaced in this context or DNA on nucleosomes being more labile and accessible for the intasome. Several factors interacting with chromatin were found to interact with IN such as the widely reported INI1 [21] and LEDGF/p75 [26], [54], [55] in addition to other proteins [56], [57]. Such a factor could compensate the natural anti-integration property of the stable chromatin, hence allowing the IN enzyme to accommodate the physical constraints within this region. This is supported by the inefficiency reported here of the physiological concerted integration reaction in a region where nucleosomes are dense and stable. Transcription and nucleosome remodeling factors are good candidates for this function because they help the DNA wrapped around histones to become accessible for integration. Since the SWI/SNF complex contains INI1 and exerts chromatin remodeling activity in the cell, we focused our work on the effect of this complex on integration using our new *in vitro* concerted integration assay into chromatin.

The use of purified SWI/SNF complexe in our integration assays with a nucleosome substrate clearly showed that efficient remodeling allows the recovery of efficient FSI (see Figure 4). More importantly, the sequencing of the integration loci in the presence of the SWI/SNF complex indicated that it specifically targeted the integration events towards the stably associated nucleosome region in an ATP-dependent manner, demonstrating that the chromatin remodeling activity of the complex was required for the process. This was confirmed by the fact that the isolated INI1, which was still able to bind IN but without catalyzing the remodeling activity, was not sufficient for restoring integration into this PN.

The targeting of integration into chromatin region I after SWI/SNF remodeling could be due to several causes. A decrease in the DNA accessibility in region II or a better accessibility of region I versus region II after remodeling were ruled out by restriction analysis (data not shown). Since the main difference between region I and region II is the stability and organisation of the nucleosomes these properties should explain the favoured integration in the region I if a direct coupling occurs. The previously reported direct IN•INI1 interaction [21], the interaction between IN and the SWI/SNF complex demonstrated here by *in vitro* co-immunoprecipitation (Figure 5A) and the requirement for this interaction in the restoration process support this hypothesis. Our results led us to propose a direct targeting of IN to the remodeled loci or an interaction between the IN and SWI/SNF-enriched region I.

This is supported by the recent demonstration that IN and INI1 also interact in a cellular context [58]. In order to answer the question about the role of INI1 in the mechanism of restoration, we tested two active remodeling human factors that don't bind directly to IN. We first tested the SNF2H subunit of the ACF-SNF2H complex, for its effect on integration into the PN templates. As reported in Figures 5C no interaction was detected *in vitro* between HIV-1 IN and SNF2H. In addition SNF2H was found able to remodel our PN template with the same efficiency than SWI/SNF (see Figure S10). However, despite this remodeling activity, no restoration of integration was detected on the PN treated by SNF2H (Figure 5D). We also analyzed the effect of BRG1, the active subunit of SWI/SNF, lacking the integrase binding cofactor INI1, but having similar remodeling efficiency as the whole complex. As reported in the Figure. 6A, despite an efficient remodeling of the template, no restoration of integration was detected.

Taken together our data indicate that the direct interaction between IN and the whole active SWI/SNF complex is required for the integration into stably associated PN. Even if the involvement of the INI1•IN interaction in the process remains to be proved the literature data as well and our own data suggest that INI1 could be the bridge for the coupling between the integration and chromatin remodeling, at least in *in vitro* integration assays. To our knowledge this is the first direct evidence of functional coupling between chromatin remodeling and retroviral integration.

According to this model the region I should constitute a preferred environment for integration after remodeling by SWI/SNF. The DNA accessibility is not sufficient to explain this process since, after remodeling, the region I was still found less accessible than the region II in REA analyses (data not shown). Thus, the simplest explanation for the integration restoration appears an enrichment of the region I with active SWI/SNF complexes due to the less dynamic and more stable properties of the nucleosomes in this domain. However the accurate mechanism of region I remodeling by SWI/SNF is not known and, more generally, the remodeling processes are still under debate in the field. Further structural analyzes of the active HIV-1 intasome complexed to a nucleosome would bring very useful information about the way nucleosome and intasome fit to accommodate with the physical constraints of this chromatin structure.

The involvement of the SWI/SNF complex in HIV-1 replication was previously studied mainly using RNA silencing approaches. Those studies suggest that INI1 could participate in several steps of the retroviral biological including nuclear import of the PIC, integration, transcription and virion production [24], [43], [58]. Several studies also showed that INI1 is not necessary for retroviral replication, but the efficiency of silencing of the INI1 genes as well as their impact on the entire SWI/SNF complex remain unclear. The latter point is crucial since it has been reported that the *in vitro* effect of INI1 on integration can vary from stimulation to inhibition depending of its concentration. The precise function of this factor in the replication cycle remains unclear so the cellular situation requires further analysis.

*In silico* comparison of 41 435 integration loci previously selected in infected cells [12] with nucleosome occupancy prediction using the method described by Milani et al. [39] also supports our *in vitro* data. Actually this model provides very similar performance in predicting the sequence dependent nucleosome positioning as the recent one developed by Field et al. [59] which corresponds to a significant improvement of the former Segal model [60]. As demonstrated by Tillo D. et al. [61], the Segal model indeed poorly accounts for the nucleosome positioning profile as observed in Yeast and other species. This new powerful predictive algorithm based on the combination of atomic force microscopy and theoretical modeling demonstrated the existence of major sequence signaling *in vivo* which involves high-energy barriers locally inhibiting nucleosome formation [39]. Analysis of the cellular integration loci showed that they were disfavored in dense and stable nucleosome regions, as observed in region I of our *in vitro* PN templates. Even if several hypotheses can explain this apparent selectivity in the cell, these data are consistent with the results obtained in our *in vitro* studies system and correlate with the integration refractory property of stable chromatin found in this model.

The cellular situation has been more widely studied by massive analysis of retroviral DNA integration with a pyrosequencing method [12], [19], [20]. In those studies, computational prediction of nucleosome positions in target DNA indicated that integration was particularly promoted near transcription-associated histone modifications, including H3 acetylation, H4 acetylation and H3 K4 methylation, but was not promoted in regions rich in transcription-inhibiting modifications, which include H3 K27 trimethylation and DNA CpG methylation. These data are consistent with our *in vitro* observation and suggest that the functional link between HIV-1 IN and cellular chromatin remodeling complexes described *in vitro* can also occur in the infected cells. Such functional interactions between both viral integration and cellular chromatin remodeling complexes are not limited to HIV-1. A strongly biased target-site selection was found for yeast Ty3 retrotransposons. Ty3 integration is promoted at the 5′ ends of RNA polymerase-III-transcribed genes via interaction between IN and RNA polymerase III transcription factors [62]. More striking examples are found for yeast TY1 retrotransposons integration, where chromatin remodeling is associated with the selectivity of integration [63], [64]. Ty1 inserts into an approximately 700-bp integration window upstream of tRNA genes approximately every 80 bp. ATP-dependent chromatin remodeling by Isw2 upstream of tRNA genes leads to changes in chromatin structure and Ty1 integration site selection. These data could indicate that these mechanisms could be more widespread in the field of viral DNA mobility.

**Figure 8:**
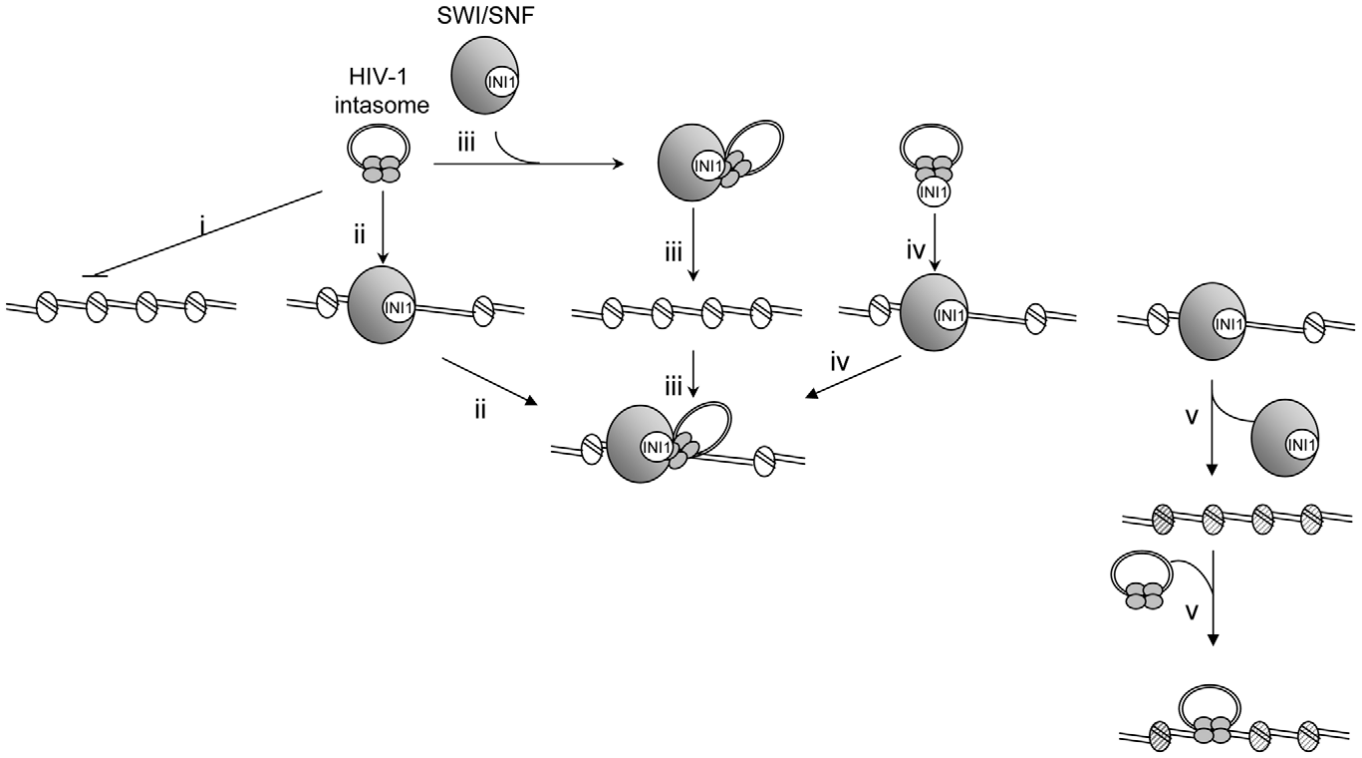
Model for HIV-1 integration into stable chromatin regions. HIV-1 integration appears disfavored in stably associated and dense nucleosome regions both *in vitro* and *in vivo* (**i**). However, functional interaction between IN and the SWI/SNF remodeling activity could allow the integration into the nucleosome-dense region of the chromatin either by serving as chromatin receptor for the intasome (**ii**) either by targeting the intasome into region of the host DNA to be remodeled (**iii**). Early interaction between IN and INI1, including in virions, could make it possible to direct the intasome in the same SWI/SNF-enriched region (**iV**). All these non-exclusive ways could lead to the formation of the same integration-remodeling complex for remodeling the integration loci and thus either to accommodate the intasome with the structural constraints of the nucleosome or to allow integration into a local nucleosome-free segment. Finally, SWI/SNF remodeling of the nucleosome structure could lead to the formation of an integration-promoted structure (hatched nucleosomes) and target the integrase and/or catalytic activity of integrase on the PN template (**V**).

Our data lead us to propose an integration/remodeling coupling model presented in Figure 8. Early interaction between the incoming viral preintegration and the SWI/SNF remodeling complexes could favor a targeted integration into actively remodeled chromatin which would thus constitutes preferential sites (Figure 8, path iii). An alternative mechanism could be the interaction between the integration complex and the SWI/SNF-enriched regions of the genomes under remodeling (Figure 8, path ii). Previous works reported the early interaction between IN, viral DNA and INI1 as well as the incorporation of this factor into HIV-1 virions [24], [43]. This is consistent with a role of INI1 in the early events of replication and with the possible early interaction between the factor and the incoming PIC, leading both to nuclear entry and targeting to the SWI/SNF-enriched region of the host DNA (Figure 8, path iv). All these non-exclusive ways could lead to the formation of a bifunctional chromatin remodeling-integration complex. Alternatively, remodeling of the nucleosome structure by the whole active SWI/SNF complex associated with activation of transcription could promote the integration steps by promoting a chromatin structure compatible for integration (DNA bulges, histone modifications etc…) (Figure 8, path v).

The exact structure of DNA in the remodeled integration site remains to be determined in order to elucidate whether integration takes place in DNA bulges formed around the nucleosome after remodeling or in naked DNA obtained after sliding of the nucleosome or ejection of the histone octamer. Our data are not inconsistent with a preferential integration into nucleosomes as shown by the previous wild range analyses of cellular integration loci reported by Bushman, but they also reveal that HIV-1 integration preferentially into a more labile region of the genome where the DNA structure can be more easily adapted during remodeling to fit with the intasome constraints for efficient integration. This model is also compatible with the involvement of additional cellular chromatin-binding factors, such as LEDGF/p75 or other transcription factors that could refine the control of integration targeting. The *in vitro* system reported here should allow extensive analysis of the impact of such factors in both the specificity and selectivity of HIV-1 integration into chromatin.

## Materials and Methods

### Proteins

HIV-1 IN standard purification was performed as previously described [35]. Purification of SWI/SNF was done as previously reported [47]. Nuclear extracts were prepared from a HeLa S3 cell line expressing Flag-tagged INI1 as described before [65]. Affinity purification of the eptitope-tagged SWI/SNF complex was performed from these extracts as described previously [46]. Flag-tagged INI1 and BRG1 were produced in SF9 cells using a baculovirus overexpression system and purified on M2-agarose beads according to previously described protocols [66]. The purity of these proteins was checked by SDS-PAGE and the remodeling activity of Flag-BRG1 was controlled by REA on a linear PN template assembled on the 5S-G5E4 fragment and on the pBSK-zeo-5S-G5E4 vector. Flag tagged SNF2H was a kind gift from G. Narlikar (UCSF, USA) and prepared as described previously [49].

### DNA substrates

Both target and pBSK-zeo donor plasmids and the 296 bp unprocessed donor were described before [35]. The 2.56 kb 5S-G5E4 fragment DNA for polynucleosome assembly (PN) was previously described [34]. It is made of two times five repeats of 5S sequences surrounding a central sequence containing five gal4 DNA binding sites and the adenovirus 2 E4 minimal promoter. We constructed a new circular acceptor template (Figure S1A) by cloning the 5S-G5E4 fragment into the pBSK-zeo vector at *Kpn*I and *Sac*I positions. Polynucleosome templates were assembled with purified HeLa core histones [67] by gradient salt dialysis [47] or using the Acf1/ISWI dependent “Chromatin Assembly Kit” from MILLIPORE following the manufacturer protocol. Structure of regions I and II was checked by *ab initio* prediction of nucleosome occupancy throughout the DNA sequence performed by computing the free-energy landscape associated with the bending of DNA around histone octamers to form nucleosomes (Figure 1C). The mathematical method is described in detail in [40], [68]. Nucleosome assembly on the vector was checked by mono- and di-nucleosome gel shift (S1B) as performed before [34] and REA assay [69] as described [50] (Figure 1D).

### Concerted integration

Standard concerted integration reactions were performed as described previously [35] using purified HIV-1 IN (12 pmoles), 5′-end-labeled donor DNA (10 ng) and circular target DNA plasmids pBSK-zeo or pBSK-zeo-5S-G5E4 (100 ng). A typical integration reaction is described in Figure S2. After reaction, integration products were loaded on 1% agarose gel, dried and autoradiographied. Quantification of the integration activity was performed using the Image J software with the following procedure: the bands corresponding to the free substrate (S), the donor/donor (d/d), linear FSI (FSI) and circular HSI+FSI (HSI+FSI) were quantified. The percentage of HSI+FSI integration activity was determined as (HSI+FSI)/[(FSI)+(HSI+FSI)+(d/d)+(S)] ×100. Percentage of FSI integration activity was determined as (FSI)/[(FSI)+(HSI+FSI)+(d/d)+(S)] ×100. Previous analyses showed that the linear FSI products was be representative of the circular FSI form, thus it was used to determine the global FSI activity. FSI reaction was additionally quantified by cloning the integration products into bacteria using the same protocol as described previously [51]. Briefly, after concerted integration, the products were purified on a DNA purification system column (Promega) as described by the supplier and then introduced into a MC1060/P3 *E. coli* strain which contained ampicillin-, tetracycline- and kanamycin-resistance genes. Both ampicillin- and tetracycline-resistance genes carry an *amb* mutation. These proteins are thus expressed only in the presence of *supF* gene products. Integration clones carrying the *supF* gene were therefore selected in the presence of 40 µg/ml ampicillin, 10 µg/ml tetracycline and 15 µg/ml kanamycin. The integration loci determination was performed by isolating plasmids from quadruple-resistant colonies and PCR sequencing (ABI Prism big dye terminator cycle sequencing ready reaction kit, Applied Biosystems) using the U_3_ primer (5′-TATGGAAGGGCTAATTCACT-3′) and the U5 primer (5′-TATGCTAGAGATTTTCCACA-3′). Only the integration products containing the 5 bp repeats flanking the integrated DNA were reported for localization in the acceptor DNA sequence.

### *In vitro* chromatin remodeling

Remodeling of the PN templates was performed with purified human SWI/SNF complex or with the BRG1 or SNF2H enzymes. 0.8, 0.6 or 1 pmoles of respectively purified SWI/SNF complex, BRG1 or SNF2H enzymes were incubated with 100 ng of acceptor DNA for 0 to 120 minutes at 30°C in a 10 µl reaction volume of buffer containing 2 mM ATP, 2 mM free MgCl_2_, 1 mM DTT, 10 mM Hepes, pH 7.5, 0.1 mM EDTA, 60 mM KCl. Remodeling was checked by REA assay [69] as described [50]. For coupling to concerted integration the recombinant IN (12 pmoles) was added to the remodeling reaction with the donor DNA (10 ng) and the complexes were formed for 20 minutes at 0°C. The integration reaction buffer was then added to reach the optimal IN activity (20 mM HEPES, pH 7.5; 10 mM DTT; 10 mM MgCl_2_; 15% DMSO; 8% PEG, 30 mM NaCl) and the reaction was carried out as described above. Remodeling activity was measured by REA assays [69] as described [50]. Assays were done in a buffer containing 12 mM Hepes (pH 7,9), 60 mM KCl, 1 mM DTT, 12% glycerol, 0.1 mM EDTA and 2 mM ATP in presence of 10 to 15 units of *Hha*I enzyme at 30°C. Typically 4 nM of DNA substrate were used in the final reaction with 0.8 pmoles of SWI/SNF for 120 minutes. The reaction was stopped by digestion with 1 mg/ml of proteinase K in presence of SDS and EDTA at 37°C for 30 min. The samples were then submitted to phenol/isoamyl alcohol/chlorophorm (24/1/25 v/v/v) extraction and analyzed on 1% agarose gels.

### *In vitro* co-immunoprecipitation

12 pmoles of pure recombinant integrase was mixed with either 0.2 pmoles of SWI/SNF complex, 6.2 pmoles of FLAG tagged INI1 or 2,7 pmoles of SNF2H protein and incubated at 37°C for 1 hour. Then rabbit polyclonal anti-BAF57/SMARCE1 (α-BAF57) (Abcam ab70540), mouse monoclonal anti-FLAG (α-FLAG) (Sigma Aldrich F1804) or rabbit polyclonal anti-SNF2h (α-SNF2H) (Abcam ab72499) antibodies or no antibodies were added to the mixture at 4°C over night. Following a 1 h 30 incubation with BSA-saturated sheep anti-rabbit/mouse IgG magnetic beads (Invitrogen Dynabeads M-280) at 4°C, the samples were washed two times with an excess of PBS BSA 1%. The samples were subjected to SDS-PAGE electrophoresis after addition of Urea-SDS buffer. The membranes were immunoblotted with either α-BAF57, α-FLAG, α-SNF2H or rabbit polyclonal anti-integrase antibodies (Bio Products MD, AB-INT 100), and HRP-conjugated secondary antibodies

### Nucleosome occupancy prediction

Nucleosome occupancy prediction was determined using the method previously described by [39].

## Supporting Information

Figure S1 Structure of the pBSK-zeo-5S-G5E4 chromatinized acceptor DNA. The position of the restriction sites used in REA assays are reported in the pBSK-zeo-5S-G5E4 sequence (A). *Eco*R1 restriction was used to control the structure of the nucleosome 5S-G5E4 domain on 0.8% agarose gel. We report the 5S mononucleosome (5S MN), GSE4 431 bp DNA and 5S 196 bp fragment in addition to polynucleosome fragments (PN) and pBSK-zeo DNA vector backbone positions of the corresponding bands for each restriction analysis of the 1/1, 1/1.33, 1/1.67 and 1/2 polynucleosomial pBSK-zeo-5S-G5E4 in addition to the naked corresponding pBSK-zeo-5S-G5E4 vector. Agarose gel shift structure analysis performed after *Eco*RI restriction one set of acceptor DNA is shown in (B).
(0.67 MB TIF)

Figure S2 Standard *in vitro* concerted integration assay. Standard concerted integration reactions were performed as described previously using purified HIV-1 IN (12 pmoles), 5′-end-labeled donor DNA (100 ng) and circular receptor DNA plasmids pBSK-zeo. The donor DNA contains 20 terminal base pair derived from the viral U3 and U5 end. The receptor DNA contains a *SupF* gene suppressing the *amb* mutation under the dependence of the bacterial EM7 promoter. This is used for selecting integrants in the MC1060/P3 E. coli strain which contained ampicillin- and tetracycline-resistance genes carrying the amb mutation. IN was incubated 20 minutes at 4°C with both the donor and the receptor DNA before adding the reaction mixture (20 mM HEPES, pH 7.5; 10 mM DTT; 10 mM MgCl_2_; 15% DMSO; 8% PEG, 30 mM NaCl) in a final volume of 10 µl. The reaction is proceeded for 90 min at 37°C. Incubation was stopped by adding a phenol/isoamyl alcohol/chloroform mix (24/1/25 v/v/v). The aqueous phase was loaded on a vertical 1% agarose gel in the presence of 1% bromophenol blue and 1 mM EDTA. After separation of the products, the gel was treated with 5% TCA for 20 min, dried and autoradiographed. After reaction three types of products are detected: donor/donor products corresponding to the strand transfer of one viral end from one donor molecule to another one, circular half site (HSI) products corresponding to the strand transfer of one viral end from one donor molecule to a circular acceptor plasmid, circular full site (FSI) products corresponding to the strand transfer of two viral ends from the same donor molecule to a circular acceptor plasmid and linear FSI corresponding to the strand transfer of two viral ends from two independent donor molecules to a circular acceptor plasmid leading to its linearization. The circular FSI and HSI can not be distinguished on gel but the circular FSI can be specifically cloned into bacteria and sequenced allowing its specific quantification and the determination of both the structure of the integrated DNA and its localization into the target DNA. Here are reported the products detected on agarose gel after reaction performed without IN (lane 1), without acceptor DNA (lane 2) and in presence of all the constituents of the reaction (lane 3).
(0.36 MB TIF)

Figure S3 Effect of nucleosome assembly on structure of integration loci. A concerted integration assay was performed with 12 pmoles of IN and 100 ng of donor DNA and 10 ng of naked pBSK-zeo-5S-G5EA plasmid (Naked), or polynucleosomal pBSK-zeo-5S-G5E4 assembled with increasing amounts of histones expressed in DNA/histones mass ratio (µg/µg) (1/1, 1/33, 1/167, 1/2). The circular FSI products were specifically quantified by cloning in bacteria and reported as the number of ampicillin-, kanamycin- and tetracycline-resistant selected clones. 100 FSI products obtained after integration in each condition were sequenced by PCR (ABI Prism big dye terminator cycle sequencing ready reaction kit, Applied Biosystems) using the U3 primer (5′-TATGGAAGGGCTAATTCACT-3′) and the U5 primer (5′-TATGCTAGAGATTTTCCACA-3′). The number of correct 5 bp duplications or other events (including other duplications or deletions) found at the extremity of the integrated DNA was reported. Not enough integrants were selected with the 1/2 PN plasmid (nd).
(0.08 MB TIF)

Figure S4 Effect of nucleosome assembly on *in vitro* HIV-1 integration into pBSK-zeo acceptor plasmid. A concerted integration assay was performed with 12 pmoles of IN and 100 ng of donor DNA and 10 ng of naked pBSK-zeo acceptor plasmid lacking the 5S-G5E4 sequence assembled with increasing amounts of mass ratios (µg/µg) of DNA/histones (lanes 1/1, 1/33, 1/1.67, 1/2). The reaction products were loaded on 1% agarose gel (A). The position and structures of the donor substrate and different products obtained after half-site (HSI), full-site (FSI) and donor/donor integration (d/d) are shown. The different integration products were quantified by densitometric estimation of the FSI and HSI+FSI heterointegration bands with the Image J software (B). The circular FSI products were specifically quantified by cloning in bacteria and reported as the number of ampicillin-, kanamycin- and tetracycline-resistant selected clones (C). All the values correspond to the mean ± standard deviation (error bars) of three independent sets of experiments.
(0.65 MB TIF)

Figure S5 Effect of Acf1/ISWI assembled nucleosomes on concerted integration. Acf1/ISWI assembly was performed in presence of recombinant histone chaperone NAP-1 and topoisomerase following the manufacturer protocol (MILIPORE). Assembly on the pBSK-zeo-5S-G5E4 vector was checked by REA assay (a typical representative experiment is reported in A). A concerted integration assay was then performed with 12 pmoles of IN and 100 ng of donor DNA and 10 ng of pBSK-zeo-5S-G5EA plasmid (Naked), or polynucleosomal pBSK-zeo-5S-G5E4 assembled with increasing amounts of histones expressed in DNA/histones mass ratio (µg/µg) (1/0.2, 1/0.6). The reaction products were loaded on 1% agarose gel and the different integration products were quantified by densitometric estimation of the FSI and HSI+FSI heterointegration bands with the Image J software (B).
(0.13 MB TIF)

Figure S6 Effect of DNA relaxation on *in vitro* integration into naked or polynucleosomal acceptor template. A concerted integration assay was performed with 12 pmoles of IN and 100 ng of donor DNA and 10 ng of naked pBSK-zeo-5S-G5EA plasmid (Naked), or polynucleosomal pBSK-zeo-5S-G5E4 after treatment of not with topoisomerase I. Relaxation was checked by agarose gel analysis after proteinase K and Phenol-chloroforme-isoamyla alcohol (24/25/1, v/v/v) treatment (an example of analysis is shown in the bottom of panel A). The reaction products were loaded on 1% agarose gel and the FSI heterointegration product was quantified with the Image J software. An example of results obtained with the naked and 1/33 PN is shown in the top of panel (A) and quantification means ± standard deviation (error bars) of three independent sets of experiments performed with the set of plasmids assembled with increasing amounts of histones expressed in DNA/histones mass ratio (µg/µg) (1/1, 1/33, 1/167, 1/2) are shown in (B). OC: relaxed open circular form of the plasmid, CC: compacted closed circular form of the plasmid.
(0.34 MB TIF)

Figure S7 Effect of nucleosome assembly on integration loci distribution in pBSK-zeo-5S-G5EA acceptor plasmid. A concerted integration assay was performed with 12 pmoles of IN and 100 ng of donor DNA and 10 ng of naked pBSK-zeo-5S-G5EA plasmid (Naked), or polynucleosomal pBSK-zeo-5S-G5E4 assembled with a 1/1.67 DNA/histones mass ratio. The circular FSI products were cloned bacteria and were sequenced for each condition. 20 correct integration loci (IL) were localized in the naked pBSK-zeo-5S-G5EA (A) and polynucleosomal pBSK-zeo-5S-G5E4 (B) vector sequence.
(0.32 MB TIF)

Figure S8 Effect of chromatin remodeling activity of SWI/SNF complex on *in vitro* integration in nucleosomal templates. Naked or chromatinized pBSK-zeo-5S-G5EA vectors assembled with increasing amounts of histones expressed in DNA/histones mass ratio (µg/µg) (1/1, 1/33, 1/167, 1/2) were treated with or without SWI/SNF in presence or not of ATP. The remodeling efficiency was controlled in a REA assay using *HhaI* restriction enzyme. The percentage of cleavage is shown for each condition in (A). A concerted integration assay was performed with 12 pmoles of IN and 100 ng of donor DNA and 10 ng of naked pBSK-zeo-5S-G5EA plasmid (Naked), or polynucleosomal pBSK-zeo-5S-G5E4 assembled with increasing amounts of histones expressed as DNA/histones mass ratio (µg/µg (1/33 and 1/2) after 0 to 120 min of SWI/SNF treatment in presence of ATP. The reaction products were loaded on 1% agarose gel (B). The position of the donor substrate and different products obtained after half-site (HSI), full-site (FSI) and donor/donor integration (d/d) are shown. The circular FSI products obtained with all the set of chromatinized plasmids after SWI/SNF treatment in presence of ATP were cloned into bacteria 100 integration loci were sequenced. The number of correct 5 bp duplications or other events (including other duplications or deletions) found at the extremity of the integrated DNA are shown in (C).
(0.42 MB TIF)

Figure S9 Effect of chromatin remodeling activity of SWI/SNF complex on integration loci distribution in pBSK-zeo-5S-G5EA acceptor plasmids. A concerted integration assay was performed with 12 pmoles of IN and 100 ng of donor DNA and 10 ng of naked pBSK-zeo-5S-G5EA plasmid (Naked) (A), or polynucleosomal pBSK-zeo-5S-G5E4 assembled with a 1/1.67 DNA/histones mass ratio and treated with SWI/SNF complex in presence (B) or not of ATP (C). The circular FSI products were cloned bacteria and were sequenced for each condition, and 20 correct integration loci (IL) were localized in the vector sequence.
(0.47 MB TIF)

Figure S10 *In vitro* remodeling activity of human SNF2H on pBSK-zeo-5S-G5E4 vectors. Naked or chromatinized pBSK-zeo-5S-G5EA vectors assembled by salt dialysis with increasing amounts of histones expressed in DNA/histones mass ratio (µg/µg) (1/1, 1/33, 1/167, 1/2) were treated with or without SNF2H in presence or not of ATP. The remodeling efficiency was controlled in a REA assay using *HhaI* restriction enzyme. The percentage of cleavage is shown for each condition. The result of a typical experiment is shown.
(0.13 MB TIF)
