# Supplementary figures and images for: Functional Coupling between HIV-1 Integrase and the SWI/SNF Chromatin Remodeling Complex for Efficient in vitro Integration into Stable Nucleosomes
Source: PLoS Pathog. 2011 Feb 10;7(2):e1001280. doi: 10.1371/journal.ppat.1001280 (PMC3037357; doi:10.1371/journal.ppat.1001280)

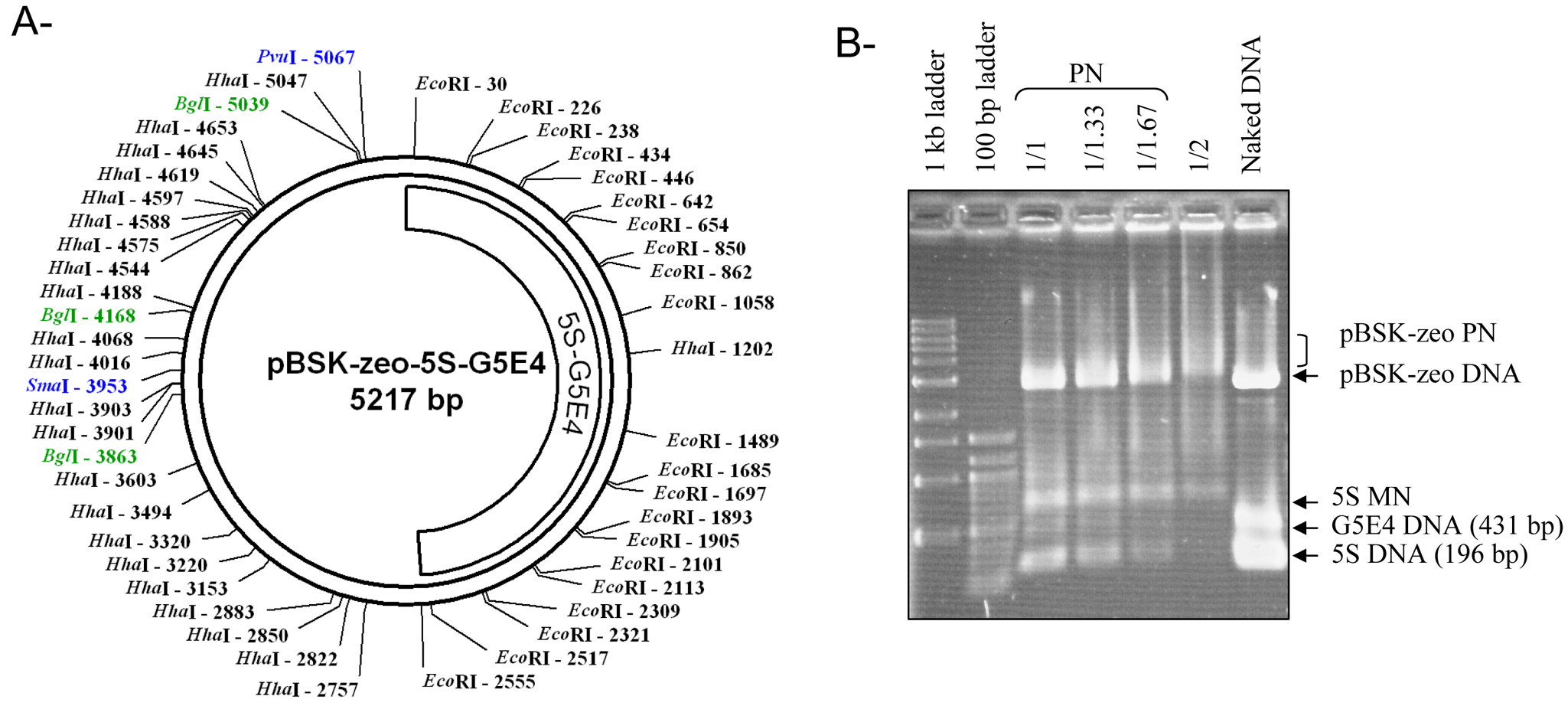

Supplement: Figure S1 — Structure of the pBSK-zeo-5S-G5E4 chromatinized acceptor DNA. The position of the restriction sites used in REA assays are reported in the pBSK-zeo-5S-G5E4 sequence (A). EcoR1 restriction was used to control the structure of the nucleosome 5S-G5E4 domain on 0.8% agarose gel. We report the 5S mononucleosome (5S MN), GSE4 431 bp DNA and 5S 196 bp fragment in addition to polynucleosome fragments (PN) and pBSK-zeo DNA vector backbone positions of the corresponding bands for each restriction analysis of the 1/1, 1/1.33, 1/1.67 and 1/2 polynucleosomial pBSK-zeo-5S-G5E4 in addition to the naked corresponding pBSK-zeo-5S-G5E4 vector. Agarose gel shift structure analysis performed after EcoRI restriction one set of acceptor DNA is shown in (B). (0.67 MB TIF) [file ppat.1001280.s001.tif]

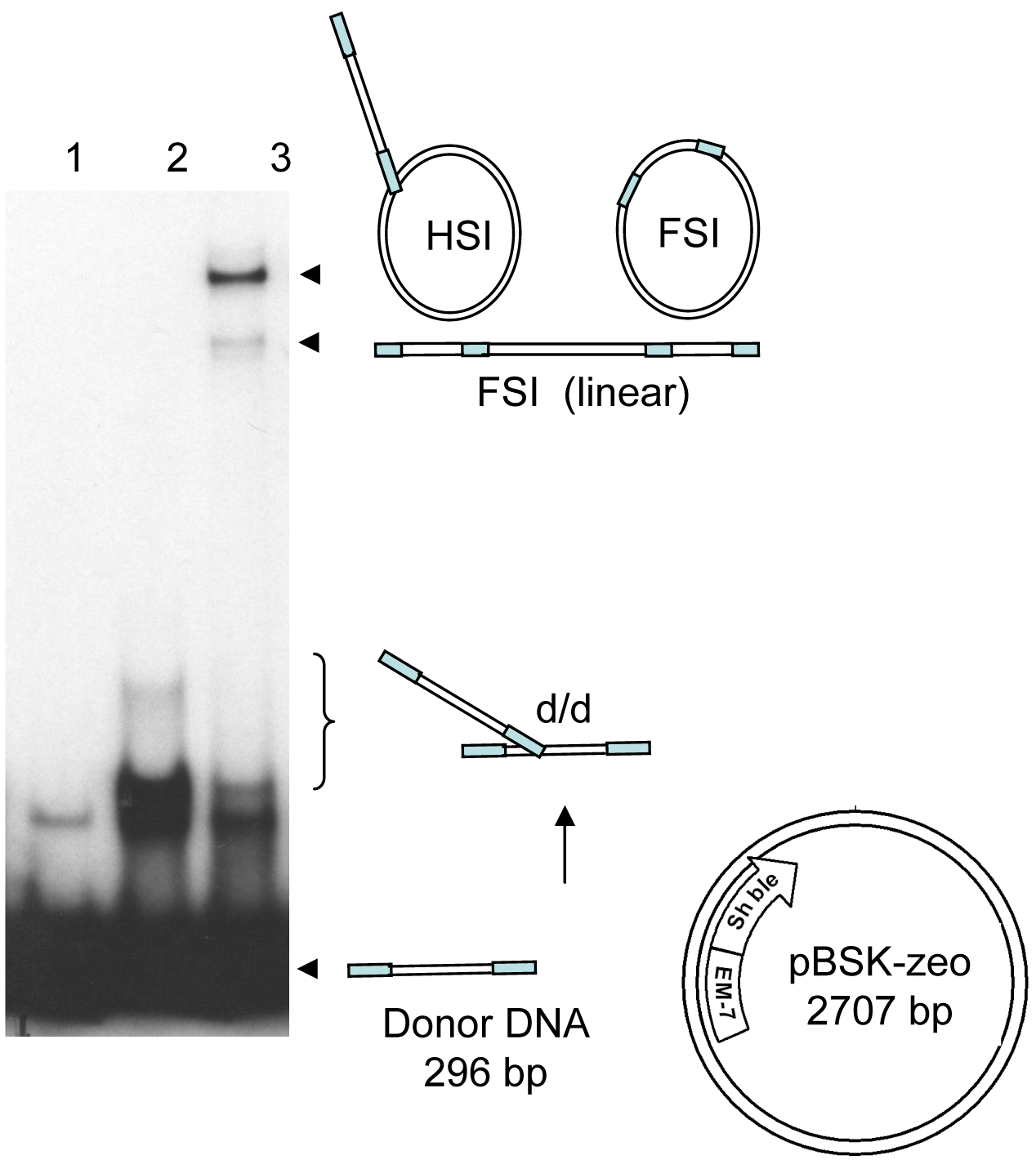

Supplement: Figure S2 — Standard in vitro concerted integration assay. Standard concerted integration reactions were performed as described previously using purified HIV-1 IN (12 pmoles), 5′-end-labeled donor DNA (100 ng) and circular receptor DNA plasmids pBSK-zeo. The donor DNA contains 20 terminal base pair derived from the viral U3 and U5 end. The receptor DNA contains a SupF gene suppressing the amb mutation under the dependence of the bacterial EM7 promoter. This is used for selecting integrants in the MC1060/P3 E. coli strain which contained ampicillin- and tetracycline-resistance genes carrying the amb mutation. IN was incubated 20 minutes at 4°C with both the donor and the receptor DNA before adding the reaction mixture (20 mM HEPES, pH 7.5; 10 mM DTT; 10 mM MgCl2; 15% DMSO; 8% PEG, 30 mM NaCl) in a final volume of 10 µl. The reaction is proceeded for 90 min at 37°C. Incubation was stopped by adding a phenol/isoamyl alcohol/chloroform mix (24/1/25 v/v/v). The aqueous phase was loaded on a vertical 1% agarose gel in the presence of 1% bromophenol blue and 1 mM EDTA. After separation of the products, the gel was treated with 5% TCA for 20 min, dried and autoradiographed. After reaction three types of products are detected: donor/donor products corresponding to the strand transfer of one viral end from one donor molecule to another one, circular half site (HSI) products corresponding to the strand transfer of one viral end from one donor molecule to a circular acceptor plasmid, circular full site (FSI) products corresponding to the strand transfer of two viral ends from the same donor molecule to a circular acceptor plasmid and linear FSI corresponding to the strand transfer of two viral ends from two independent donor molecules to a circular acceptor plasmid leading to its linearization. The circular FSI and HSI can not be distinguished on gel but the circular FSI can be specifically cloned into bacteria and sequenced allowing its specific quantification and the determination of bot [file ppat.1001280.s002.tif]

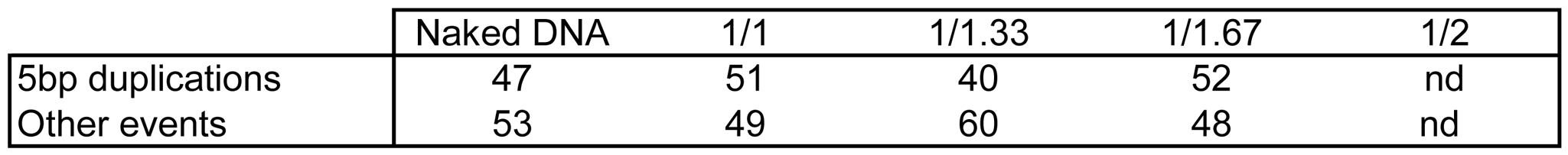

Supplement: Figure S3 — Effect of nucleosome assembly on structure of integration loci. A concerted integration assay was performed with 12 pmoles of IN and 100 ng of donor DNA and 10 ng of naked pBSK-zeo-5S-G5EA plasmid (Naked), or polynucleosomal pBSK-zeo-5S-G5E4 assembled with increasing amounts of histones expressed in DNA/histones mass ratio (µg/µg) (1/1, 1/33, 1/167, 1/2). The circular FSI products were specifically quantified by cloning in bacteria and reported as the number of ampicillin-, kanamycin- and tetracycline-resistant selected clones. 100 FSI products obtained after integration in each condition were sequenced by PCR (ABI Prism big dye terminator cycle sequencing ready reaction kit, Applied Biosystems) using the U3 primer (5′-TATGGAAGGGCTAATTCACT-3′) and the U5 primer (5′-TATGCTAGAGATTTTCCACA-3′). The number of correct 5 bp duplications or other events (including other duplications or deletions) found at the extremity of the integrated DNA was reported. Not enough integrants were selected with the 1/2 PN plasmid (nd). (0.08 MB TIF) [file ppat.1001280.s003.tif]

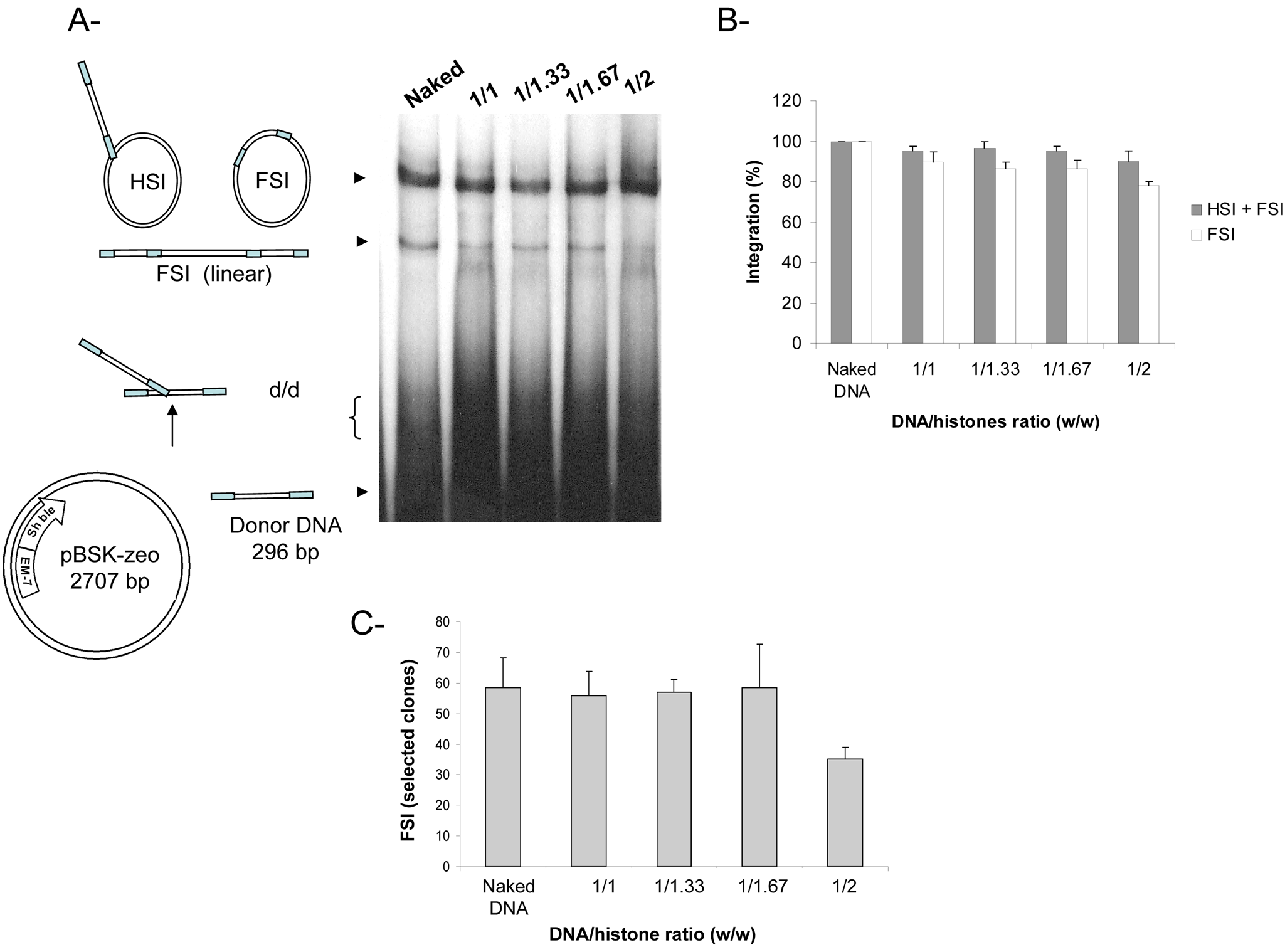

Supplement: Figure S4 — Effect of nucleosome assembly on in vitro HIV-1 integration into pBSK-zeo acceptor plasmid. A concerted integration assay was performed with 12 pmoles of IN and 100 ng of donor DNA and 10 ng of naked pBSK-zeo acceptor plasmid lacking the 5S-G5E4 sequence assembled with increasing amounts of mass ratios (µg/µg) of DNA/histones (lanes 1/1, 1/33, 1/1.67, 1/2). The reaction products were loaded on 1% agarose gel (A). The position and structures of the donor substrate and different products obtained after half-site (HSI), full-site (FSI) and donor/donor integration (d/d) are shown. The different integration products were quantified by densitometric estimation of the FSI and HSI+FSI heterointegration bands with the Image J software (B). The circular FSI products were specifically quantified by cloning in bacteria and reported as the number of ampicillin-, kanamycin- and tetracycline-resistant selected clones (C). All the values correspond to the mean ± standard deviation (error bars) of three independent sets of experiments. (0.65 MB TIF) [file ppat.1001280.s004.tif]

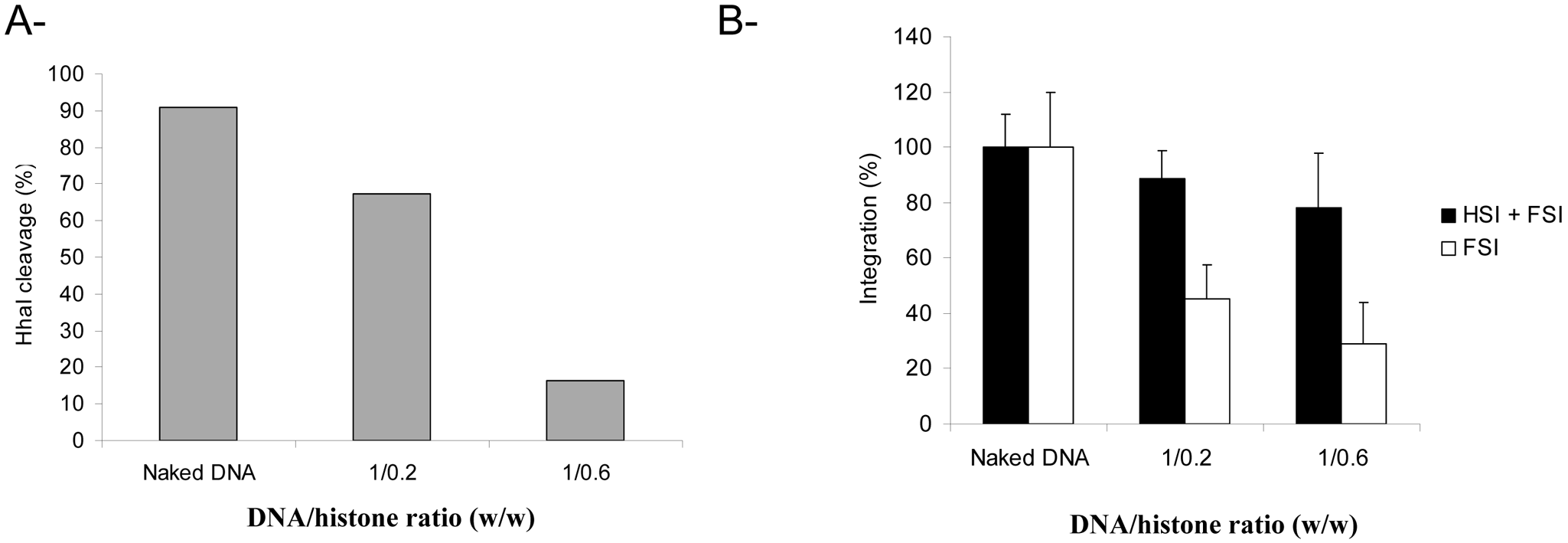

Supplement: Figure S5 — Effect of Acf1/ISWI assembled nucleosomes on concerted integration. Acf1/ISWI assembly was performed in presence of recombinant histone chaperone NAP-1 and topoisomerase following the manufacturer protocol (MILIPORE). Assembly on the pBSK-zeo-5S-G5E4 vector was checked by REA assay (a typical representative experiment is reported in A). A concerted integration assay was then performed with 12 pmoles of IN and 100 ng of donor DNA and 10 ng of pBSK-zeo-5S-G5EA plasmid (Naked), or polynucleosomal pBSK-zeo-5S-G5E4 assembled with increasing amounts of histones expressed in DNA/histones mass ratio (µg/µg) (1/0.2, 1/0.6). The reaction products were loaded on 1% agarose gel and the different integration products were quantified by densitometric estimation of the FSI and HSI+FSI heterointegration bands with the Image J software (B). (0.13 MB TIF) [file ppat.1001280.s005.tif]

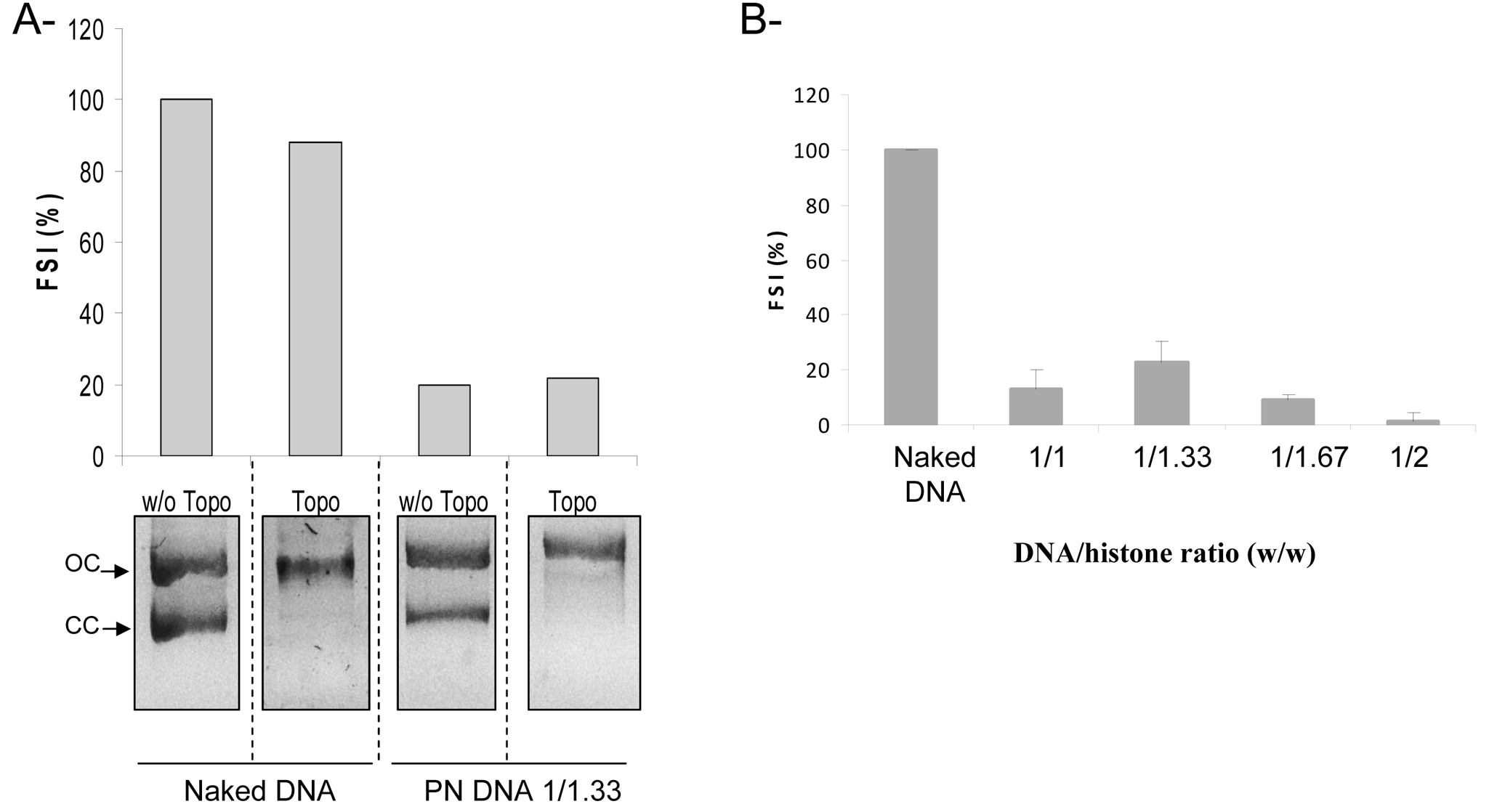

Supplement: Figure S6 — Effect of DNA relaxation on in vitro integration into naked or polynucleosomal acceptor template. A concerted integration assay was performed with 12 pmoles of IN and 100 ng of donor DNA and 10 ng of naked pBSK-zeo-5S-G5EA plasmid (Naked), or polynucleosomal pBSK-zeo-5S-G5E4 after treatment of not with topoisomerase I. Relaxation was checked by agarose gel analysis after proteinase K and Phenol-chloroforme-isoamyla alcohol (24/25/1, v/v/v) treatment (an example of analysis is shown in the bottom of panel A). The reaction products were loaded on 1% agarose gel and the FSI heterointegration product was quantified with the Image J software. An example of results obtained with the naked and 1/33 PN is shown in the top of panel (A) and quantification means ± standard deviation (error bars) of three independent sets of experiments performed with the set of plasmids assembled with increasing amounts of histones expressed in DNA/histones mass ratio (µg/µg) (1/1, 1/33, 1/167, 1/2) are shown in (B). OC: relaxed open circular form of the plasmid, CC: compacted closed circular form of the plasmid. (0.34 MB TIF) [file ppat.1001280.s006.tif]

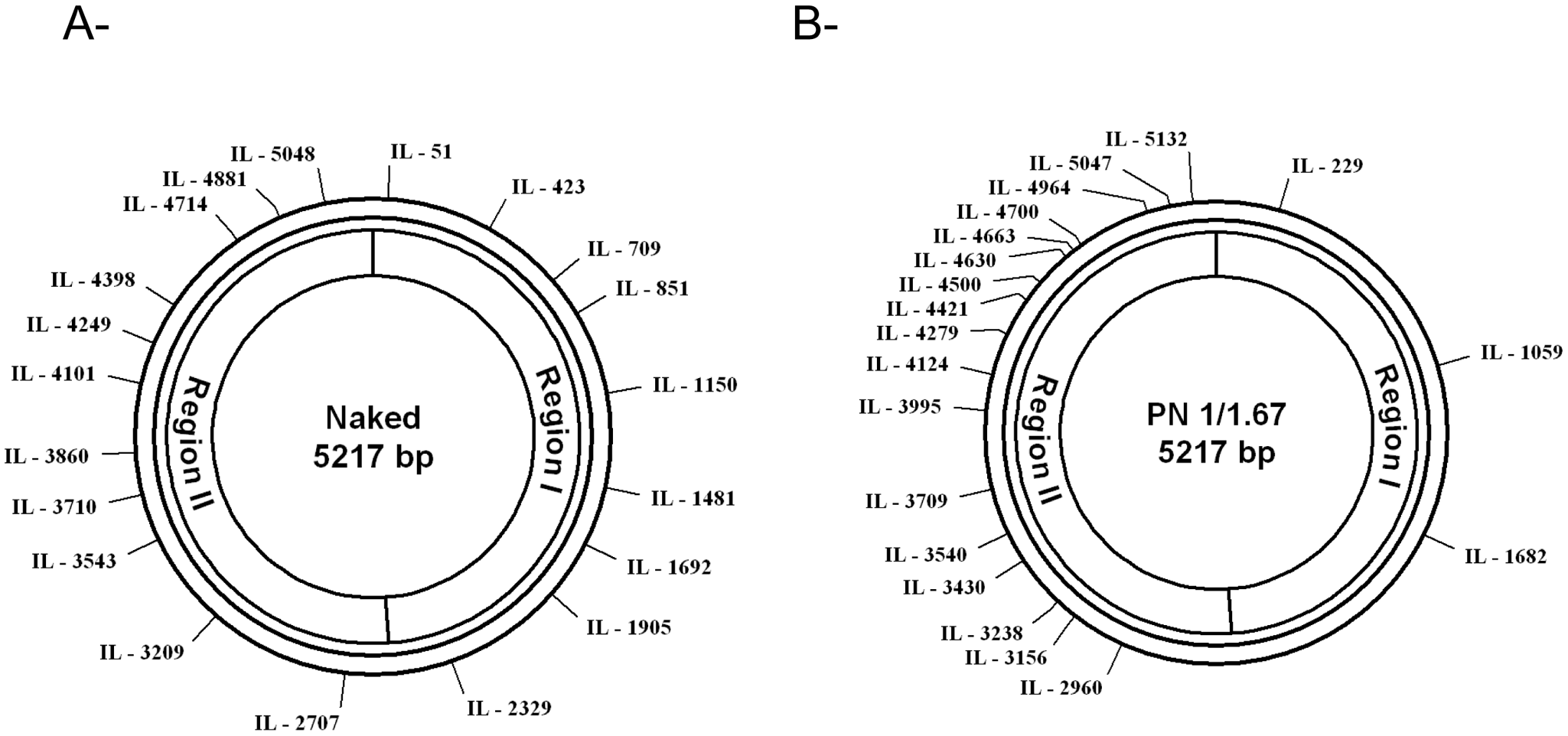

Supplement: Figure S7 — Effect of nucleosome assembly on integration loci distribution in pBSK-zeo-5S-G5EA acceptor plasmid. A concerted integration assay was performed with 12 pmoles of IN and 100 ng of donor DNA and 10 ng of naked pBSK-zeo-5S-G5EA plasmid (Naked), or polynucleosomal pBSK-zeo-5S-G5E4 assembled with a 1/1.67 DNA/histones mass ratio. The circular FSI products were cloned bacteria and were sequenced for each condition. 20 correct integration loci (IL) were localized in the naked pBSK-zeo-5S-G5EA (A) and polynucleosomal pBSK-zeo-5S-G5E4 (B) vector sequence. (0.32 MB TIF) [file ppat.1001280.s007.tif]

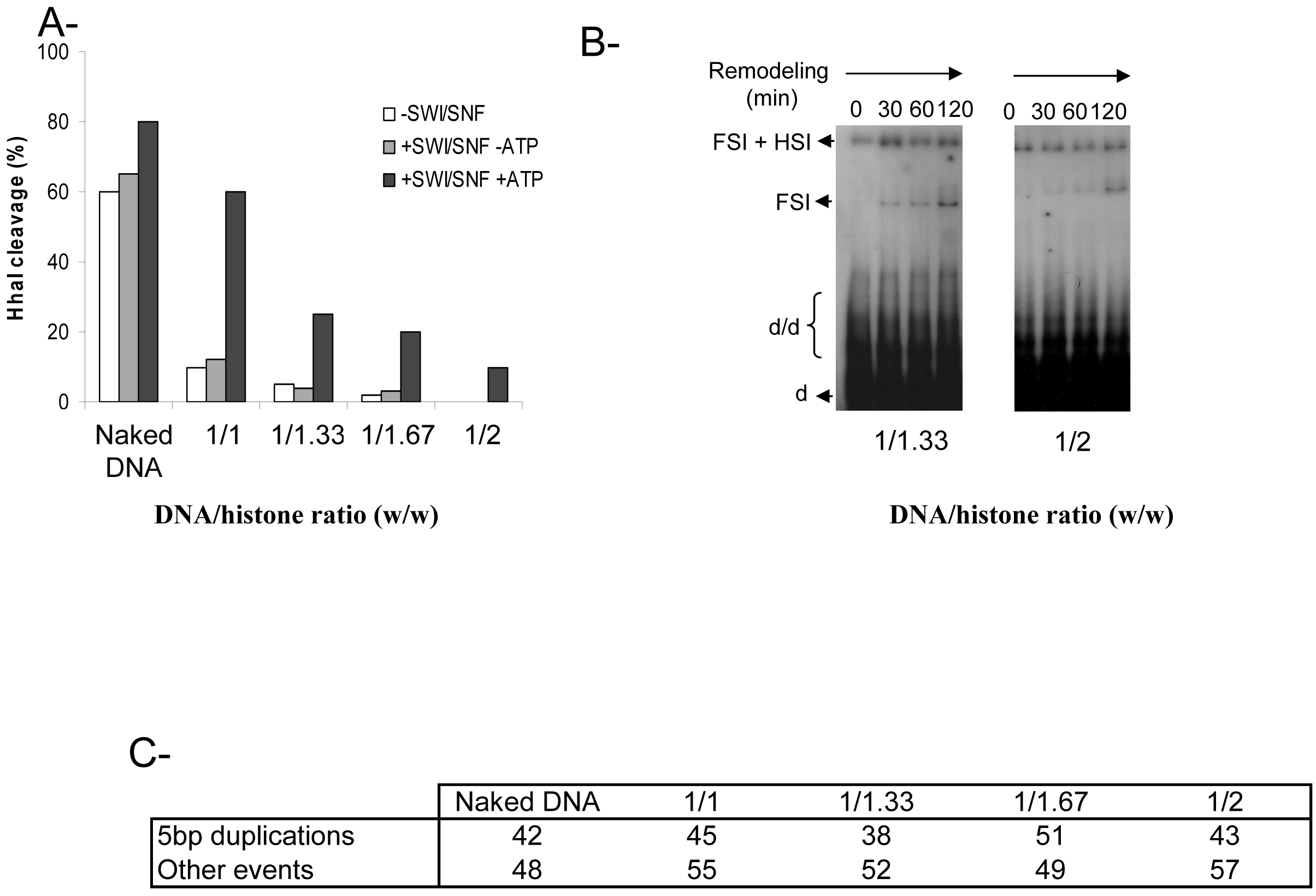

Supplement: Figure S8 — Effect of chromatin remodeling activity of SWI/SNF complex on in vitro integration in nucleosomal templates. Naked or chromatinized pBSK-zeo-5S-G5EA vectors assembled with increasing amounts of histones expressed in DNA/histones mass ratio (µg/µg) (1/1, 1/33, 1/167, 1/2) were treated with or without SWI/SNF in presence or not of ATP. The remodeling efficiency was controlled in a REA assay using HhaI restriction enzyme. The percentage of cleavage is shown for each condition in (A). A concerted integration assay was performed with 12 pmoles of IN and 100 ng of donor DNA and 10 ng of naked pBSK-zeo-5S-G5EA plasmid (Naked), or polynucleosomal pBSK-zeo-5S-G5E4 assembled with increasing amounts of histones expressed as DNA/histones mass ratio (µg/µg (1/33 and 1/2) after 0 to 120 min of SWI/SNF treatment in presence of ATP. The reaction products were loaded on 1% agarose gel (B). The position of the donor substrate and different products obtained after half-site (HSI), full-site (FSI) and donor/donor integration (d/d) are shown. The circular FSI products obtained with all the set of chromatinized plasmids after SWI/SNF treatment in presence of ATP were cloned into bacteria 100 integration loci were sequenced. The number of correct 5 bp duplications or other events (including other duplications or deletions) found at the extremity of the integrated DNA are shown in (C). (0.42 MB TIF) [file ppat.1001280.s008.tif]

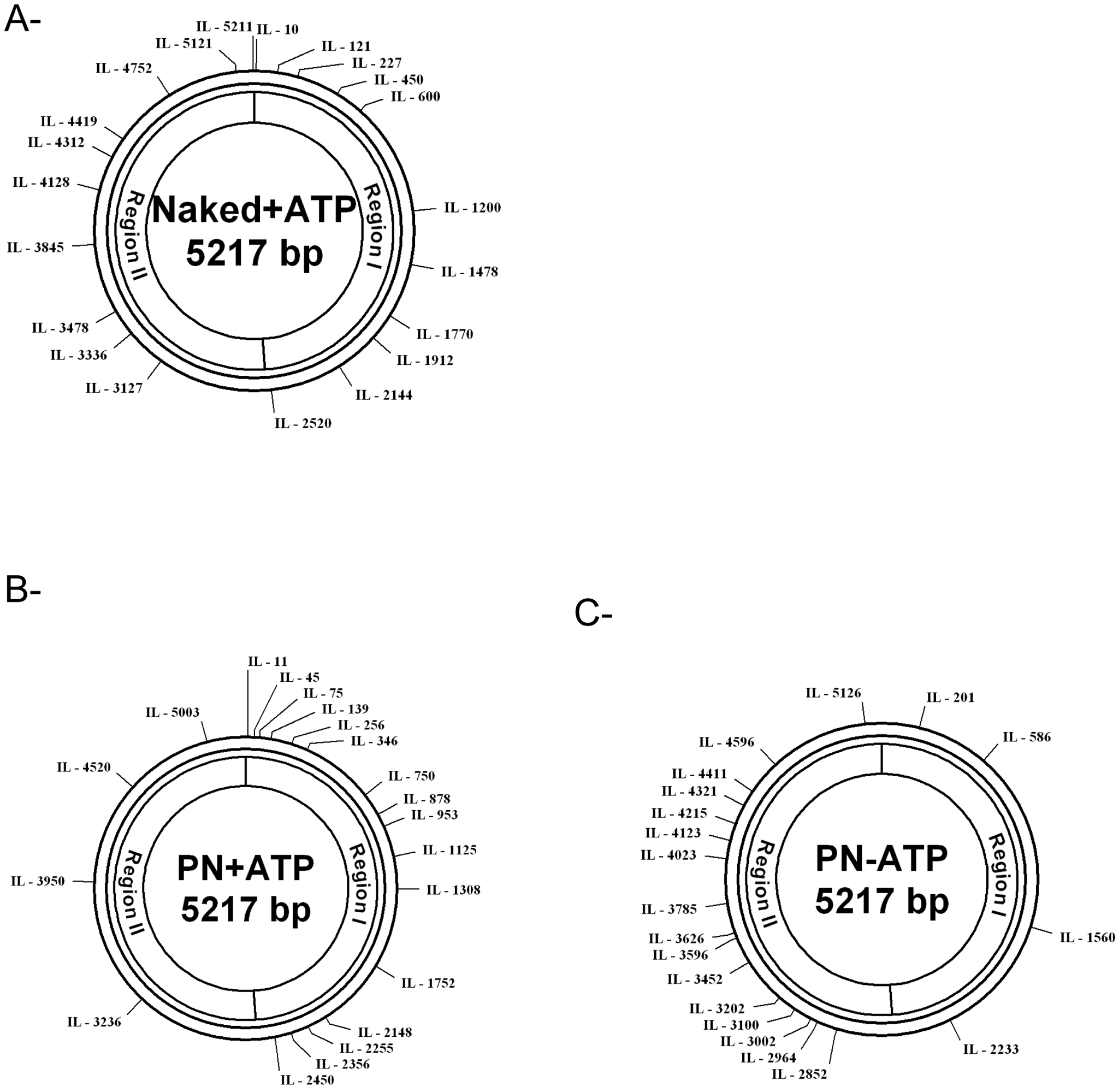

Supplement: Figure S9 — Effect of chromatin remodeling activity of SWI/SNF complex on integration loci distribution in pBSK-zeo-5S-G5EA acceptor plasmids. A concerted integration assay was performed with 12 pmoles of IN and 100 ng of donor DNA and 10 ng of naked pBSK-zeo-5S-G5EA plasmid (Naked) (A), or polynucleosomal pBSK-zeo-5S-G5E4 assembled with a 1/1.67 DNA/histones mass ratio and treated with SWI/SNF complex in presence (B) or not of ATP (C). The circular FSI products were cloned bacteria and were sequenced for each condition, and 20 correct integration loci (IL) were localized in the vector sequence. (0.47 MB TIF) [file ppat.1001280.s009.tif]

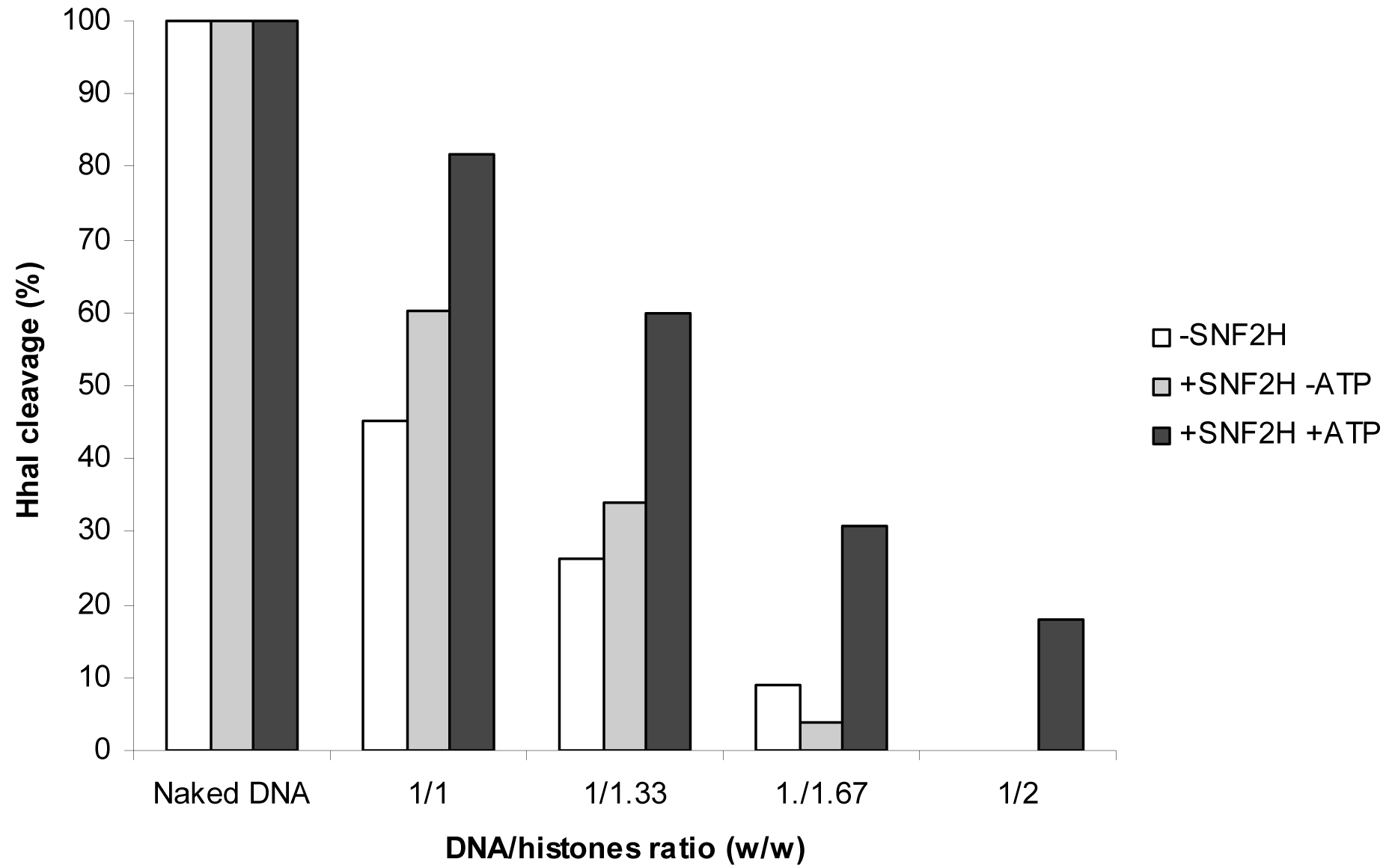

Supplement: Figure S10 — In vitro remodeling activity of human SNF2H on pBSK-zeo-5S-G5E4 vectors. Naked or chromatinized pBSK-zeo-5S-G5EA vectors assembled by salt dialysis with increasing amounts of histones expressed in DNA/histones mass ratio (µg/µg) (1/1, 1/33, 1/167, 1/2) were treated with or without SNF2H in presence or not of ATP. The remodeling efficiency was controlled in a REA assay using HhaI restriction enzyme. The percentage of cleavage is shown for each condition. The result of a typical experiment is shown. (0.13 MB TIF) [file ppat.1001280.s010.tif]
